# Supplementary material for: Realistic phase diagram of water from “first principles” data-driven quantum simulations
Source: Nat Commun. 2023 Jun 8;14:3349. doi: 10.1038/s41467-023-38855-1 (PMC10250386; doi:10.1038/s41467-023-38855-1)
Supplement: Supplementary file 1 — Supplementary Information [file 41467_2023_38855_MOESM1_ESM.pdf]

Supplementary Information

Realistic Phase Diagram of Water from "First Principles" Data-Driven Quantum Simulations

S.L. Bore and F. Paesani

Supplementary Note 1: MB-pol and DNN@MB-pol potentials

1.1 MB-pol

Since the MB-pol potential of water has already been described in detail in the literature,[1–3] we only overview here its salient features. MB-pol was derived from the many-body expansion (MBE) of the energy that allows for determining the energy, E_N, of an arbitrary system containing N (atomic or molecular) monomers as the sum of individual n-body energy contributions:

$$E_N(1,\ldots,N) = \sum_{i=1}^{N} \epsilon^{1\mathrm{B}}(i) + \sum_{i<j}^{N} \epsilon^{2\mathrm{B}}(i,j) + \sum_{i<j<k}^{N} \epsilon^{3\mathrm{B}}(i,j,k) + \cdots + \epsilon^{\mathrm{NB}}(1,\ldots,N) \tag{1}$$

Here, $\epsilon^{1\mathrm{B}}$ represents the distortion energy of an isolated monomer, such that $\epsilon^{1\mathrm{B}}(i) = E(i) - E_{\mathrm{eq}}(i)$ where $E_{\mathrm{eq}}(i)$ is the energy of the i-th monomer in its equilibrium geometry. The n-body energies, ϵ^{nB}, are defined recursively for $1 < n \leq N$ according to

$$\begin{aligned} \epsilon^{\mathrm{nB}} = E_{\mathrm{n}}(1,\ldots,n) - \sum_{i=1}^{N} \epsilon^{1\mathrm{B}}(i) - \sum_{i<j}^{N} \epsilon^{2\mathrm{B}}(i,j) - \sum_{i<j<k} \epsilon^{3\mathrm{B}}(i,j,k) - \cdots \\ \cdots - \sum_{i<j<k<\ldots}^{N} \epsilon^{(\mathrm{n}-1)\mathrm{B}}(i,j,k,\ldots,n-1) \end{aligned} \tag{2}$$

MB-pol approximates Eq. 2 as

$$E_N(1,..,N) = \sum_{i=1}^{N} \epsilon^{1\mathrm{B}}(i) + \sum_{i<j}^{N} \epsilon^{2\mathrm{B}}(i,j) + \sum_{i<j<k}^{N} \epsilon^{3\mathrm{B}}(i,j,k) + E_{\mathrm{POL}} \tag{3}$$

In MB-pol, the 1-body term (ϵ^{1B}) is represented by the potential developed by Partridge and Schwenke.[4] The 2-body term (ϵ^{2B}) describes four distinct contributions: permanent electrostatics, dispersion, 2-body polarization, and 2-body short-range interactions.[1] The 3-body term (ϵ^{3B}) describes two distinct contributions: 3-body polarization and 3-body short-range interactions.[2] The 2-body and 3-body short-range interactions are represented by 2-body and 3-body permutationally invariant polynomials (PIPs)[5] that were optimized in to reproduce 2-body and 3-body energies calculated at the CCSD(T) level of theory in the complete basis set (CBS) limit.[1,2] In the actual implementation of MB-pol,[1,2] the 2-body and 3-body polarization contributions are implicitly included in the $E_{\rm POL}$ term of Eq. 3 which represents many-body interactions at all orders through a classical polarization term. Further details of the MB-pol potential can be found in the original references.[1–3]

By correctly representing both short- and long-range many-body interactions at all orders,[6] MB-pol has been shown to accurately predict structural, thermodynamic, dynamical, and spectroscopic properties of water,[7] from gas-phase clusters[8–10] to liquid water[11–16] and ice.[17–20]

1.2 DNN@MB-pol

The deep neural network potential trained on MB-pol data (DNN@MB-pol) was developed with the smooth edition of the deep potential (DeepPot-SE) toolkit,[21] following the procedure reported in Ref. 22 which is schematically represented in Supplementary Figure 1. Specifically, we used 25, 50, and 100 neurons for the hidden embedding layers, respectively, while the submatrix of the embedding matrix uses 16 neurons. The distance cutoff was set to 6 Å, with a smoothing region of

0.5 Å. The DNN@MB-pol potential is represented by a fully connected deep neural network with three layers of 240 neurons each.

The training set for DNN@MB-pol was constructed from that of the DNN potential introduced in Ref. 24, which includes configurations collected from MB-pol simulations of supercooled water carried out at 1 atm,[16] as well as configurations selected through active learning iterations that al-

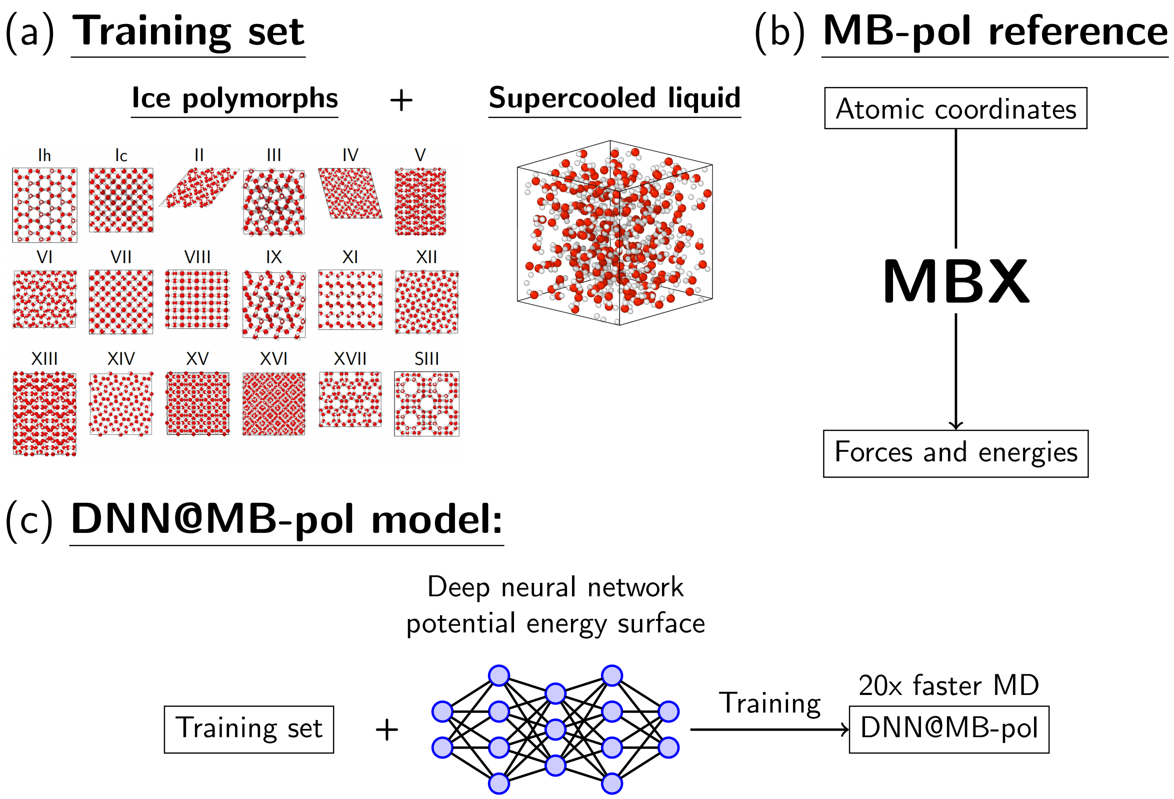

Supplementary Figure 1: **Schematic illustrating the development of the DNN@MB-pol potential.** (a) The DNN@MB-pol training set includes configurations of ice polymorphs and deeply supercooled liquid at various thermodynamic conditions extracted from MB-pol simulations as well as active learning configurations collected from previous iterations of DNN@MB-pol. (b) The labeling of the training set configurations, i.e., the calculation of MB-pol reference energies and forces, was perfomed using the MBX software.[23] (c) The DNN@MB-pol potential was trained on the training set defined in (a) using DeePMD-kit. When patched with LAMMPS, DNN@MB-pol provides a 20x speedup relative to MB-pol.

Supplementary Table 1: **Composition of the DNN@MB-pol training set.** The total dataset contains four subsets: 1) configurations extracted from MB-pol simulations of supercooled water at different pressures, 2) configurations of liquid water collected from active learning (AL) iterations in Ref. 24, 3) configurations of 18 different ice phases extracted from MB-pol simulations, and 4) configurations of 18 different ice phases extracted from two AL iterations carried out with DNN@MB-pol. The total dataset was randomly divided into training, validation, and test sets with a ratio of 0.80:0.1:0.1. During the training process, each of the four subsets was assigned a training weight of 25%.

Subset	Thermodynamic range	Number of configurations
MB-pol liquid	$1 \rightarrow 4000$ atm, $198 \rightarrow 500$ K	37755
MB-pol, 18 ice phases	$-10000 \rightarrow 100000$ atm, $100 \rightarrow 400$ K	6812
AL, Liquid	1 atm, $198 \rightarrow 368$ K	8223
AL, 18 ice phases	$-10000 \rightarrow 100000$ atm, $100 \rightarrow 400$ K	14824
Total		51203

low the DNN potential to accurately reproduce various thermodynamic properties of liquid water calculated with MB-pol at 1 atm. To guarantee full transferability of the DNN@MB-pol potential over a wider range of thermodynamic conditions, the original DNN training set was expanded by including configurations of supercooled water at high pressure, as well as configurations of 18 ice phases spanning a comprehensive set of thermodynamic conditions (Supplementary Figure 1a). The composition of the training set for the DNN@MB-pol potential is summarized in Supplementary Table 1, and the training set is deposited at https://dplibrary.deepmd.net/. MB-pol reference energies and forces for all training set configurations (Supplementary Figure 1b) were calculated using the MBX software.[23]

The DNN@MB-pol potential was trained for 4 million steps using the DeePMD-kit (Supplementary Figure 1c),[22] with a learning rate starting at 0.0005 and decreasing linearly every 5000 steps to 1.8×10^{-8}. The initial weighting factor for the energy was set to 0.2 and increased linearly to 1.0 during the training process. The initial weighting factor for the forces was set to 1000 and

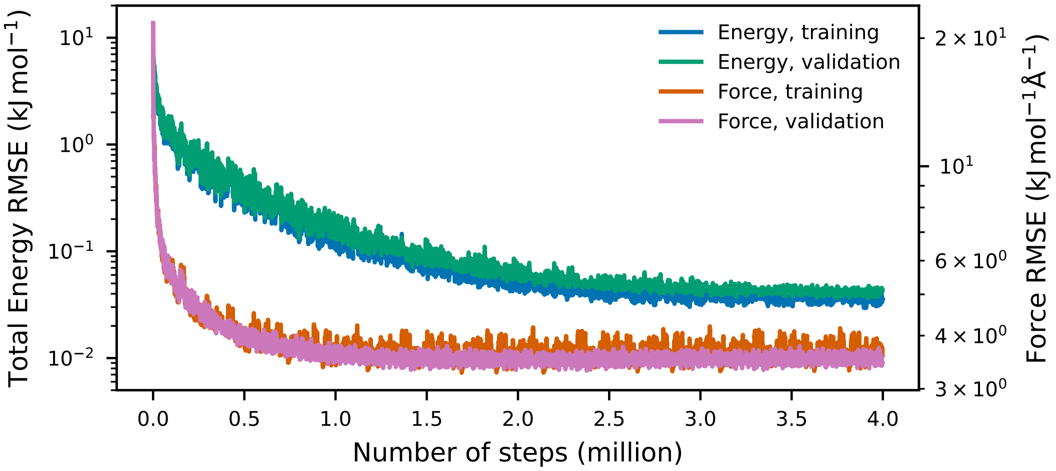

Supplementary Figure 2: **Learning curves.** Learning curves displaying the variation of the RMSEs associated with DNN@MB-pol calculations of energies and forces for configurations in the training and validation sets relative to the MB-pol reference values. For visual clarity, we show values averaged over 200 training steps.

decreased linearly to 1.0 at during the training process. The learning curves shown in Supplementary Figure 2 demonstrate that both energies and forces of the DNN@MB-pol potential reach well-behaved convergence at the end of the training process. Importantly, training and validation sets exhibit similar root-mean-square errors (RMSEs), which indicates that the DNN@MB-pol potential is not in the overfitting regime at any stage of the training process. The RMSE associated with the DNN@MB-pol forces plateaus at ~0.4 kJ mol^{-1}Å^{-1}, which is of similar magnitude to RMSEs reported for other state-of-the-art machine-learned potentials.[25]

Supplementary Figure 3 shows the RMSEs associated with DNN@MB-pol calculations for a test set containing different water systems. With the exception of ice VII and specific active learning sets, which contain molecular configurations generated from very high-pressure molecular dynamics (MD) simulations as well as highly distorted configurations, the RMSEs for the various sets of

configurations are always smaller than 0.1 kJ mol^{-1}. To put the DNN@MB-pol results in context, the most accurate DFT models of water exhibit RMSEs of 0.9 kJ mol^{-1} and 0.5 kJ mol^{-1} for absolute and relative lattice energies of different ice polymorphs, respectively.[26]

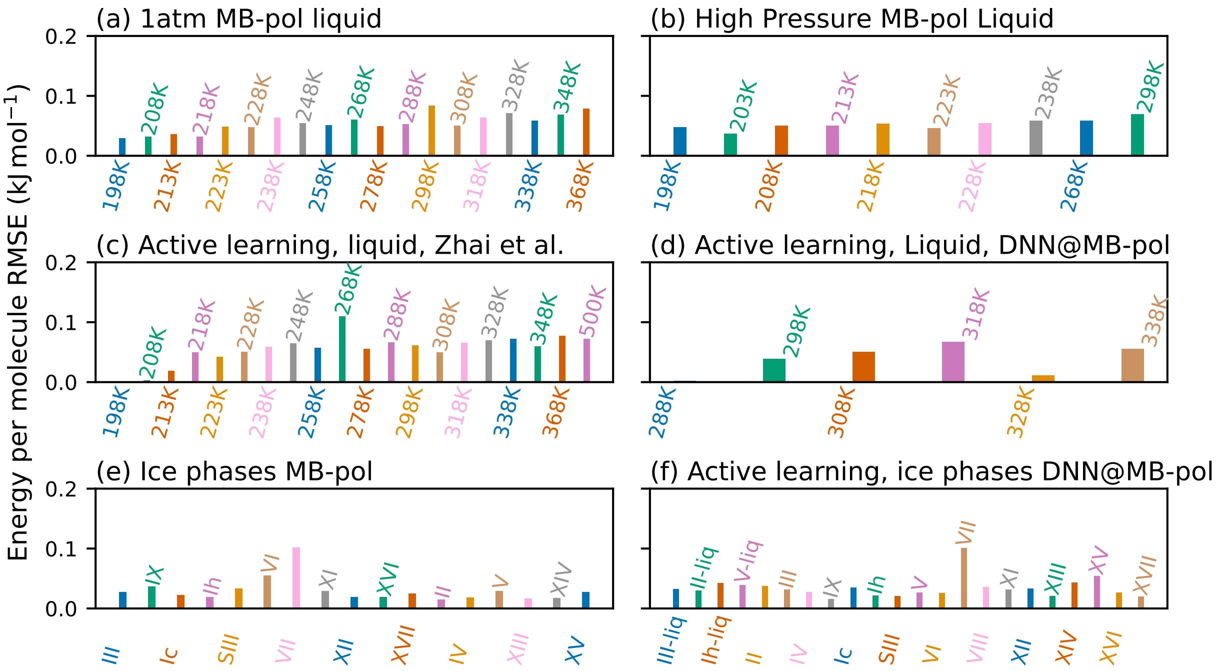

Supplementary Figure 3: **Test set errors.** RMSEs associated with DNN@MB-pol calculations of energies and forces for configurations in the test set relative to the MB-pol reference values. By definition, test set configuration were not used in training or validation.

Supplementary Note 2: Molecular dynamics simulations

The MD simulations with the DNN@MB-pol potential were carried out using LAMMPS[27] patched with the PLUMED enhanced-sampling plugin[28] and DeePMD-kit[29] with model compression.[30] DeePMD-kit with model compression provides a speedup of ∼20× relative to MD simulations carried out with the reference MB-pol potential carried out with LAMMPS patched with MBX.[23] We employed a time step of 0.5 fs for the integration of the equations of motion that were propagated according to the velocity-Verlet algorithm. Depending on the ensemble, the temperature was controlled using the stochastic velocity rescaling algorithm with a relaxation time of 0.1 ps[31] and the pressure was maintained by a Parrinello-Rahman barostat with a relaxation time of 1 ps.[32]

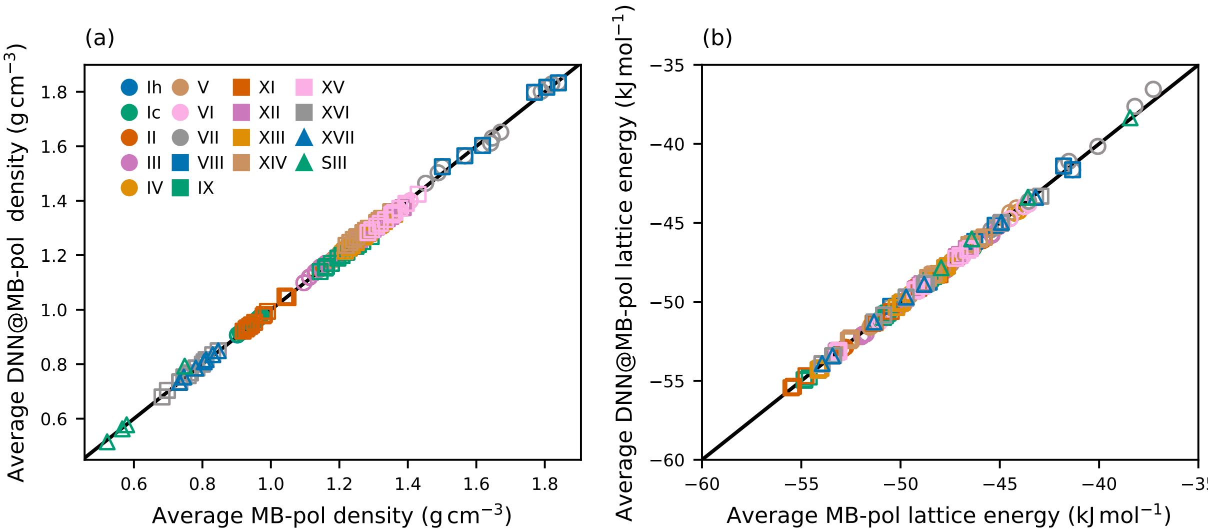

Supplementary Figure 4: **Correlation between DNN@MB-pol and MB-pol.** Correlation plots for the density (a) and potential energy (b) of liquid water calculated from NPT simulations carried out with DNN@MB-pol and the corresponding values calculated with the reference MB-pol potential.

Supplementary Figure 4 shows the correlation between the average densities and lattice energies of different ice polymorphs calculated from NPT simulations carried out with the DNN@MB-pol potential and the corresponding MB-pol values. The low RMSEs exhibited by the energies per molecule calculated with DNN@MB-pol relative to the corresponding MB-pol values for the test set shown in Supplementary Figure 3 are reflected in a quantitative agreement between DNN@MB-pol and MB-pol values for both densities and lattice energies of all ice polymorphs, indicating that DNN@MB-pol samples the same configurations as MB-pol. Importantly, as shown in Supplementary Figure 5, the ice densities predicted by DNN@MB-pol are also in excellent agreement with the corresponding experimental values.

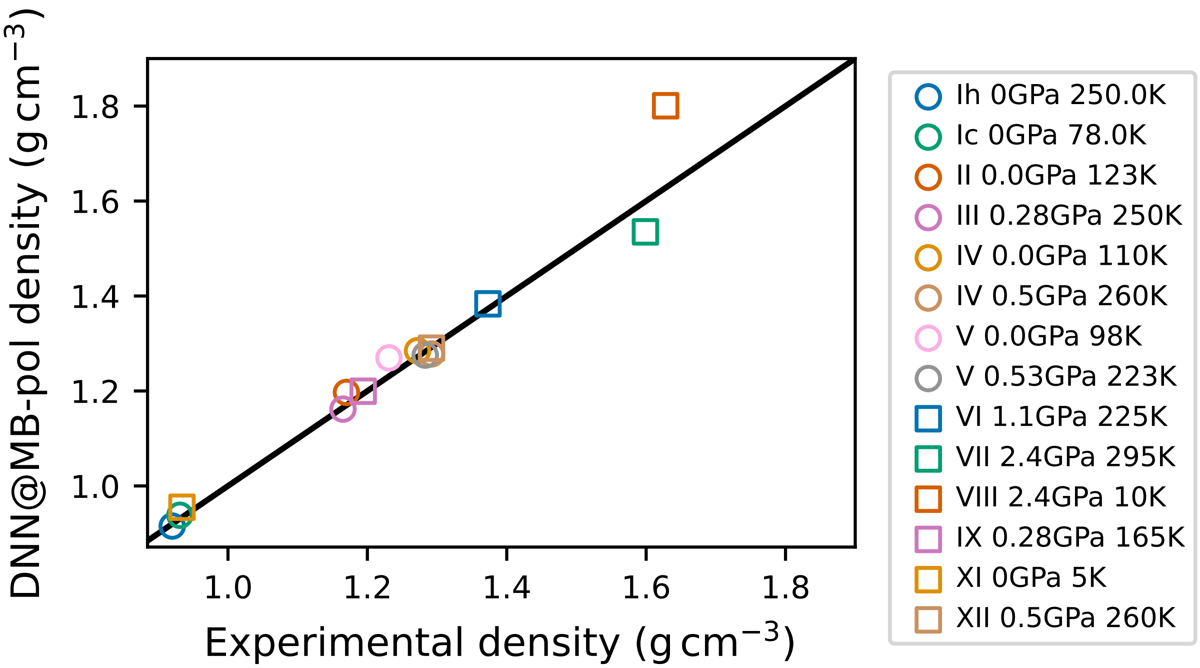

Supplementary Figure 5: **Densities of ice polymorphs.** Correlation plot between the densities of different ice polymorphs calculated with the DNN@MB-pol potential and the corresponding experimental values.[33]

Supplementary Figure 6 shows that DNN@MB-pol also closely reproduces the temperature dependence of the density and isothermal compressibility of liquid water calculated with MB-pol at 1 atm from the boiling point down to the supercooled regime.[16]

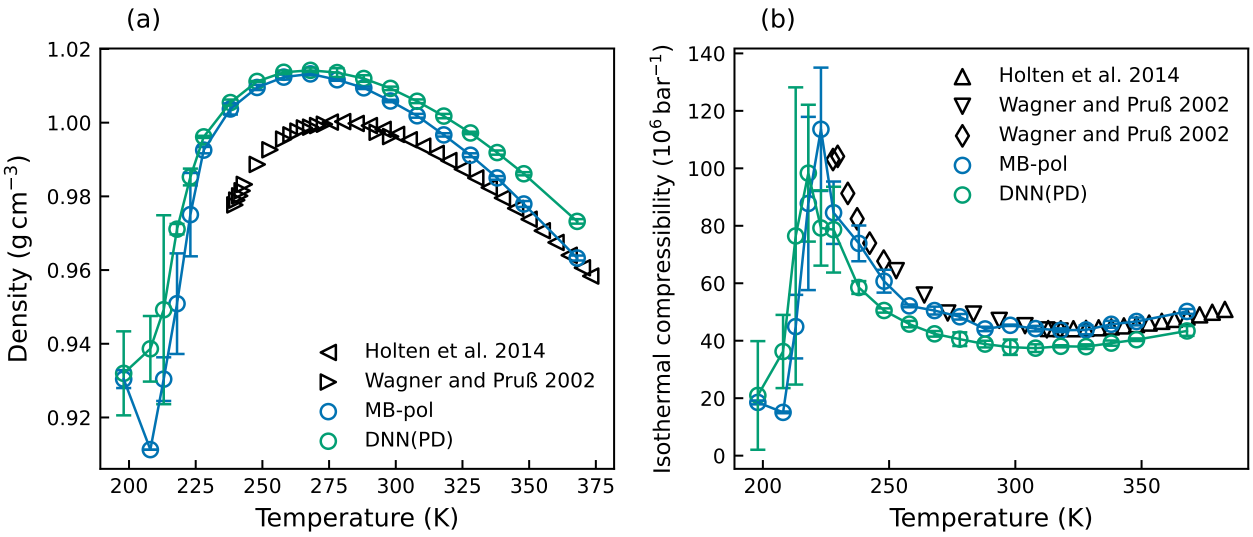

Supplementary Figure 6: **Supercooled water.** Temperature dependence of the density (a) and isothermal compressibility (b) calculated from the NPT simulations carried out with the DNN@MB-pol potential at 1 atm (green) compared to the reference MB-pol values from Ref. 16 (blue). The associated error bars indicate 95% confidence intervals of the averages. To account for the correlation between MD frames, the error bars were computed 95% confidence intervals by blocking the time series into four blocks and then multiplying the standard deviation associated with the average values calculated for each block by 1.96. Open black symbols correspond to the experimental values from Refs. 34 and 35 for the density, and Refs. 36, 37 and 38 for the isothermal compressibility.

Supplementary Note 3: Phase diagram of water: Algorithms

We calculated the phase diagram of water at the classical level using the DNN@MB-pol and MB-pol potentials, and at the quantum-mechanical level (i.e., including nuclear quantum effects) using the MB-pol potential. These calculations were carried out following a four-stage procedure as illustrated in Supplementary Figure 7: (a) we determined the classical melting points from

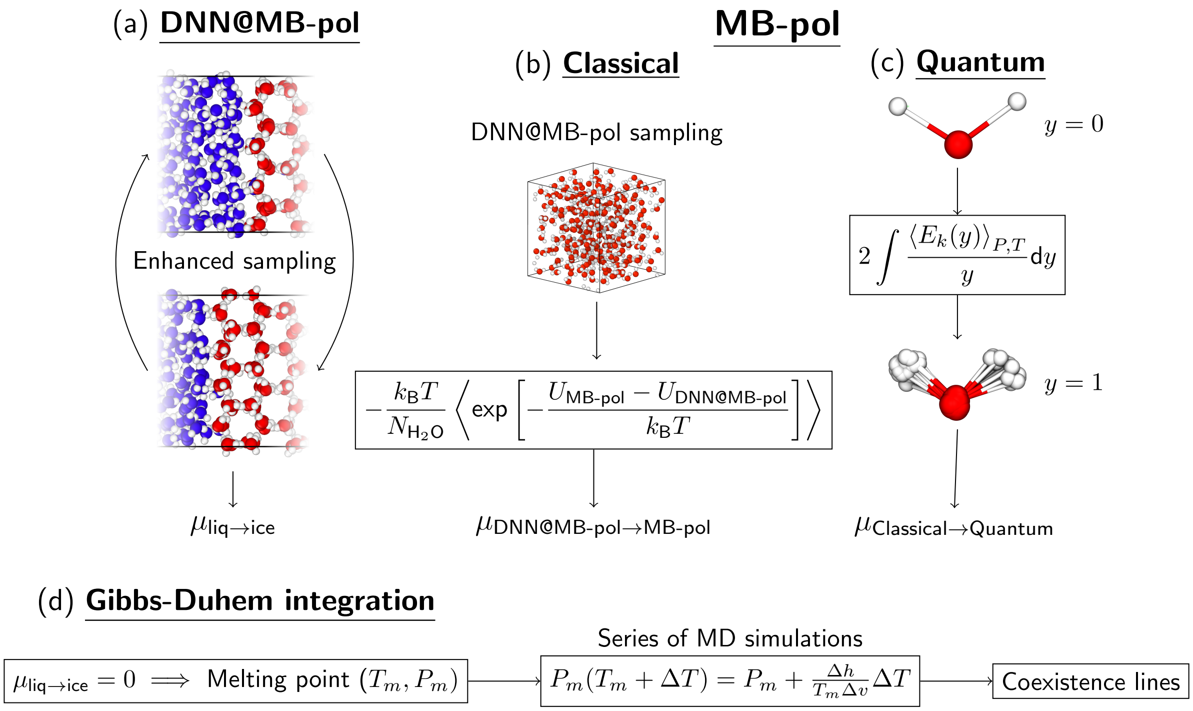

Supplementary Figure 7: **Calculations of classical and quantum chemical potentials.** (a) The differences in chemical potentials between liquid water and each ice polymorph were initially calculated using enhanced-coexistence simulations carried out with the DNN@MB-pol potential. (b) These differences in chemical potential was elevated to the corresponding classical MB-pol values by performing thermodynamic perturbation calculations. (c) The quantum corrections to the MB-pol classical chemical potentials were calculated by performing thermodynamic integration by mass within the path-integral formalism. (d) From the classical and quantum melting points of each ice polymorph calculated with the DNN@MB-pol and MB-pol potentials, we traced the corresponding coexistence lines using Gibbs-Duhem integration, for which the integrands were calculated by performing a series of MD simulations at different temperatures and pressure to determine the differences in volume, Δv, and enthalpy, Δh.

the chemical potential differences between liquid water and each ice polymorph using enhanced-coexistence simulations carried with the DNN@MB-pol potential, (b) starting from the classical melting points obtained with DNN@MB-pol, we used thermodynamic perturbation theory to determine the classical melting points of MB-pol, (c) starting from the classical melting points of MB-pol, we performed thermodynamic integration to determine the corresponding quantum melting points of MB-pol, and (d) we performed Gibbs-Duhem integration to trace the coexistence lines between the different phases and then determine the DNN@MB-pol and MB-pol phase diagrams.

3.1 Chemical potentials from enhanced-coexistence simulations

To calculate the chemical potential differences between liquid water and each of the ice polymorphs, we applied the enhanced-coexistence method introduced in Ref. 39. The enhanced-coexistence method was inspired by the interface pinning method[40] and introduces a bias potential such that the number of ice-like molecules in the system reversibly samples the number of molecules in one ice layer (Supplementary Figure 7a). This procedure allows for computing chemical potentials and, thus, coexistence points.

In the enhanced-coexistence simulations, we used the number of ice-like molecules as the order parameter, which was calculated using the "environment similarity" approach.[41] Specifically, we constructed a set of environments $X = \chi_1, ..., \chi_m$ for each ice polymorph (Supplementary Figure 8a shows an example for ice I_h). Environments were centered on the oxygen atoms. The hydrogen atoms were only included for proton-ordered ice II and not included in the environments of the other proton-disordered polymorphs to avoid any bias towards a particular configuration.[42]

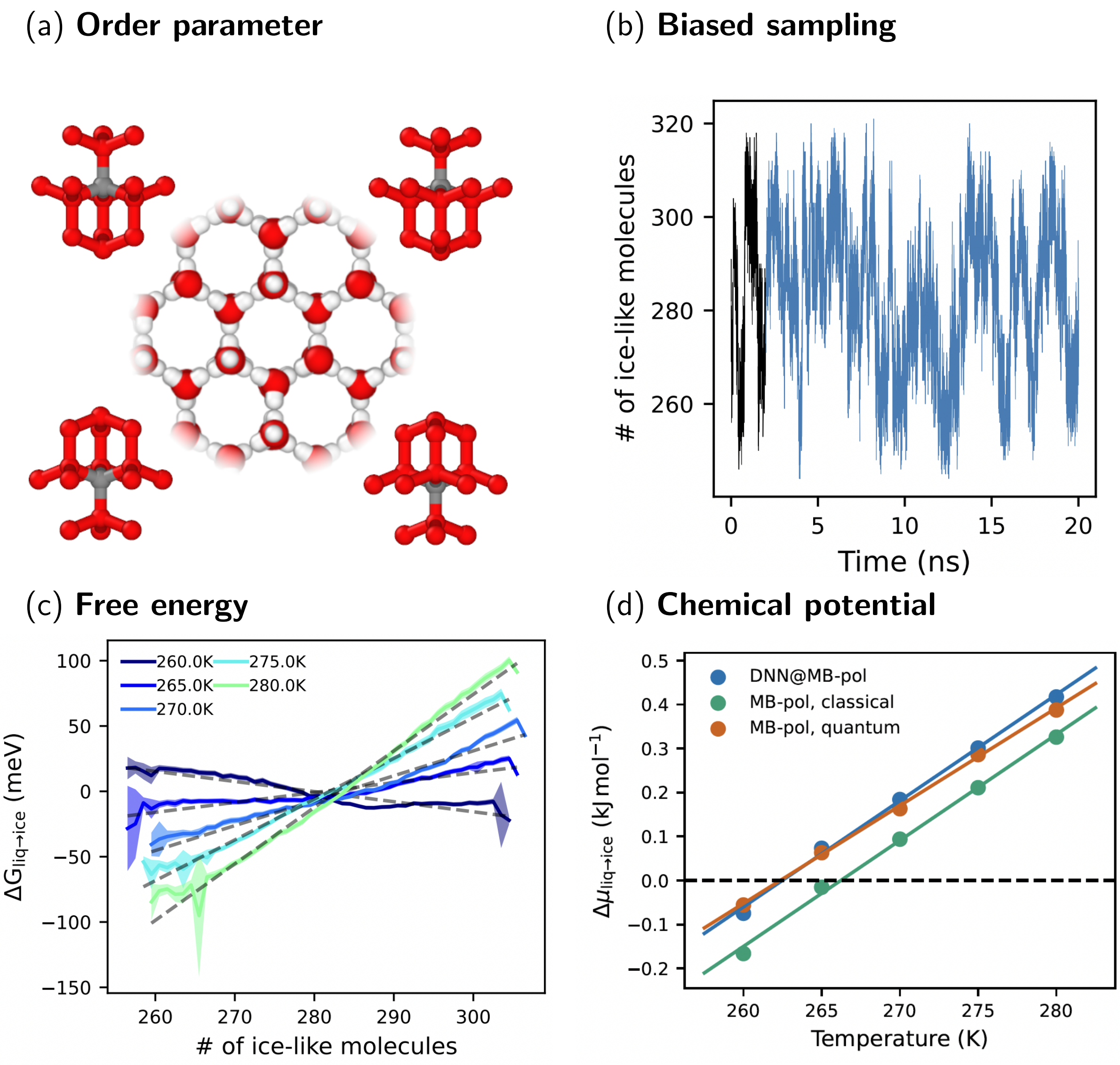

Supplementary Figure 8: **Chemical potential difference between liquid water and ice $\mathrm{I_h}$.** a) As order parameter for the enhanced-coexistence simulations we used the number of ice-like molecules defined by the "environment similarity" approach. For the case of ice Ih, four environments were used. b) We monitored the variation in number of ice-like molecules along the enhanced-coexistence trajectories. c) Using reweighting, we determined the differences in free energy ($\Delta G_{\mathrm{liq}\to\mathrm{ice}}$) between liquid water and each ice polymorph as a function of the number of ice-like molecules. d) From the slope of the free-energy differences as a function of the number of ice-like molecules, we calculated the corresponding differences in chemical potentials ($\Delta\mu_{\mathrm{liq}\to\mathrm{ice}}$) at difference temperatures. The location of the melting point of each ice polymorph was thus defined by the temperature at which the difference in chemical potential between liquid water and the ice polymorph is zero.

The "environment similarity" kernel defined as[43,44]

$$k_{\chi_l}(\chi) = \frac{1}{n}\sum_{i\in\chi_l}\sum_{j\in\chi}\exp\left(-\frac{|\mathbf{r}_i^l - \mathbf{r}_j|^2}{4\sigma^2}\right) \tag{4}$$

was then used to compare the atomic environments $\chi_l \in X$ with a generic environment χ. In Eq. 4, the atomic densities are represented by sums of Gaussians, with spread σ, centered on the neighbors' positions, n is the number of neighbors in the environment χ_l, and $\mathbf{r}_i^l$ and $\mathbf{r}_j$ are the positions of the neighbors in environments χ_l and χ, respectively. To obtain a single similarity measure between a given environment and any of the m reference environments of a given ice polymorph, we used the best-match kernel defined in Eq. 5, which compares the environments χ to all environments X of a given polymorph,

$$k_X(\chi) = \max\{k_{\chi_l}(\chi) : \chi_l \in X\}, \tag{5}$$

Since $k_X(\chi)$ as defined in Eq. 5 is not continuous and not differentiable, and, therefore, cannot directly be used in enhanced-coexistence simulations, we used the corresponding smooth version introduced in Ref. 39. The similarity kernel defined in Eq. 5 allows for determining the compatibility of the environments found in each configuration sampled during an enhanced-coexistence simulation with the environments of the target ice polymorph. For a system of N water molecules there are N oxygen environments $\chi^1, \chi^2, ..., \chi^N$. We thus defined a global order parameter n_{ice}

that represents the number of environments consistent with a given ice polymorph as

$$n_{ice} = \{\text{number of } \chi^i \ : \ k_X(\chi^i) > \kappa\}, \tag{6}$$

Here, κ is a watershed between values of $k_X(\chi^i)$ consistent with liquid water and those consistent with the target ice polymorph.

The bias potential in the enhanced-coexistence simulations was applied using the on-the-fly probability enhanced sampling (OPES) algorithm,[45,46] which shares several features with metadynamics.[47] The bias potential was defined to be a function of n_{ice} within a multi-umbrella ensemble,[46] producing an approximately uniform distribution in the interval from n_{ice}^{low} to n_{ice}^{high}. The limits of the interval were chosen as integer multiples of the number of molecules in a crystal layer, Δn_{layer}, i.e., $n_{ice}^{low} = N_l \Delta n_{layer}$ and $n_{ice}^{high} = (N_l + 1)\Delta n_{layer}$ with $N_l \in \mathbf{N}$. In the simulations, the spacing between n_{ice}^{λ} was one molecule. ΔF_{λ} was defined as the difference in free energy between the unbiased system and the system with an umbrella potential at n_{ice}^{λ}, and was determined iteratively as described in Ref. 46. In Supplementary Figure 8b, we show an example of the variation of the number of ice-like molecules along one of the water-$\mathrm{I_h}$ enhanced-coexistence simulations carried out at 270 K. After an initial transient period in which a suitable bias potential is identified, the simulation proceeds to reversibly sample all of the ice-like molecules that are targeted.

Once the bias potential had converged, we calculated the equilibrium properties of the system by

reweighting:

$$P(n_{ice}) = \frac{\langle \delta(n_{ice} - n_{ice}(\mathbf{R}))e^{\beta V} \rangle_V}{\langle e^{\beta V} \rangle_V} \tag{7}$$

Here, $\langle \cdot \rangle_V$ indicates an average over the biased ensemble, and V is the bias potential. In practice, the application of Eq. 7 corresponds to constructing a histogram with weights $e^{\beta V}$. The free-energy differences between liquid water and each ice polymorph at a given temperature T were then calculated as a function of n_{ice} as

$$\Delta G(n_{ice}) = -\frac{1}{\beta} \log P(n_{ice}). \tag{8}$$

where $\beta = 1/kT$. An example of $\Delta G(n_{ice})$ between liquid water and ice I_h is shown in Supplementary Figure 8c.

Following Ref. 39, the chemical potential difference between the liquid and each ice polymorph was determined from the slope of $\Delta G(n_{ice})$ with respect to n_{ice} over one ice layer according to:

$$\frac{\Delta G(n_{ice} + \Delta n_{layer}) - \Delta G(n_{ice})}{\Delta n_{layer}} = \mu_{ice} - \mu_{liq}. \tag{9}$$

In practice, we determined $\mu_{liq \to ice} = \mu_{ice} - \mu_{liq}$ by extracting the slope from $\Delta G(n_{ice})$ calculated using Eq. 8. The slope was obtained by performing an error-weighted linear least-squares fit. From the chemical potential differences, we calculated the melting points T_m for all ice polymorphs using the condition that $\mu_{liq \to ice}(T_m) = 0$ for liquid water and ice in equilibrium at constant pressure. T_m was obtained by an error-weighted linear least-squares fit to $\mu_{liq \to ice}$ as a function of the tem-

perature. Supplementary Figure 8d shows an example of the chemical potential difference between liquid water and ice I_h.

3.2 Thermodynamic perturbation corrections

While the comparisons shown in Supplementary Figures 3-6 demonstrate a high degree of consistency between the DNN@MB-pol and MB-pol potentials, nonnegligible differences still exist, which ultimately can manifest in shifted coexistence lines in the corresponding phase diagrams. To elevate the chemical potential differences calculated with DNN@MB-pol to the MB-pol level, we applied thermodynamic perturbation theory to determine the differences in chemical potentials between the two potentials according to[48]

$$\Delta\mu_{\mathrm{DNN@MB-pol}\rightarrow\mathrm{MB-pol}}(P,T) = -\frac{k_{\mathrm{B}}T}{N}\left\langle \exp\left[-\frac{U_{\mathrm{MB-pol}} - U_{\mathrm{DNN@MB-pol}}}{k_{\mathrm{B}}T}\right]\right\rangle_{P,T,\mathrm{DNN@MB-pol}} \tag{10}$$

Here, $\langle\rangle_{P,T,\mathrm{DNN@MB-pol}}$ denotes an ensemble average over molecular configurations of liquid water and each ice polymorph sampled by MD simulations carried out with the DNN@MB-pol potential at temperature T and pressure P for simulation boxes containing N molecules, and $U_{\mathrm{MB-pol}}$ and $U_{\mathrm{DNN@MB-pol}}$ are the MB-pol and DNN@MB-pol potential energies associated with each configuration, respectively.

3.3 Nuclear quantum effects

Due to the light mass of the hydrogen atoms, nuclear quantum effects can play an important role in determining the properties of water[49,50] and have been shown to lead to significant shifts in

the coexistence equilibria between liquid water and the ice polymorphs simulated with various water models.[48,51,52] To elevate the MB-pol differences in chemical potentials between liquid water and each ice polymorph calculated at the classical level to the corresponding quantum values, we applied thermodynamic integration by mass within the path-integral formalism to integrate the centroid virial kinetic energy, E_k, from infinitely massed molecules ($y = 0$) to the actual mass of the H_2O molecules ($y = 1$) according to[53]

$$\Delta\mu_{\text{Clas.}\to\text{NQE}}(P,T) = 2\int_0^1 \frac{\langle E_k(y)\rangle_{P,T}}{y}\mathrm{d}y, \text{where } y = \sqrt{m/\tilde{m}} \tag{11}$$

In practice, we computed the integral in Eq. 11 for all relevant (T, P) equilibrium state points between liquid water and each ice polymorph by performing path-integral molecular dynamics (PIMD) simulations[54,55] to determine E_k for different y values. After discretizing the integration interval by computing the centroid viral kinetic energy for $y = 0$, $1/4$, $1/2$, $\sqrt{2}/2$ and 1, we calculated the integral using Simpson's rule.

3.4 Gibbs-Duhem integration

We traced the coexistence lines from the calculated melting points using Gibbs-Duhem integration.[56] In practice, we numerically integrated the Clausius-Clapeyron equation:

$$\frac{\mathrm{d}P}{\mathrm{d}T} = \frac{\Delta h}{T_m \Delta v}, \tag{12}$$

where Δh and Δv are enthalpy and volume differences per molecule, respectively, between liquid water and the target ice polymorph in equilibrium, and T_m is the melting point of the target ice polymorph at pressure P. We integrated Eq. 12 using the fourth-order Runge–Kutta method,[57] evaluating the integrand for each integration step by performing one MD simulation for each phase. We determined the location of all triple points from the intersection between the relevant liquid-ice coexistence lines. We then carried out a new Gibbs-Duhem simulation for the two ice phases starting from each liquid-ice-ice triple point. We located new triple points from these coexistence lines and repeated the procedure until the entire phase diagram was computed.

Supplementary Note 4: Phase diagram of water: Simulations

4.1 Systems setup

The initial configurations for the different ice polymorphs were prepared using GENICE,[58,59] which ensures that the ice configuration has the correct proton order/disorder and zero total dipole moment, while satisfying the Bernard-Fowler ice rules.[60] Starting from the ice configurations, all relevant liquid-ice coexistence configurations were generated as follows. For ice I_h, ice III, ice V, and ice VI, the initial coexistence configurations were taken from analogous simulations carried out with the TIP4P/Ice model in Ref. 39. First, the ice box was equilibrated in the NPT ensemble with an anisotropic barostat for 1 ns. This was followed by 300 ps of simulation in the NP_xT ensemble (fluctuating box dimensions in the x-direction and constant area in the yz-directions) during which the motion of half of the water molecules on one side of the box was kept frozen, while the water molecules on the other half of the box were melted at 450 K. The whole system was then equilibrated at 270 K and 1.0133×10^{-4} GPa, 230 K and 0.303 GPa, 250 K and 0.5066 GPa, 300 K and 1.0133 GPa for ice I_h, ice III, ice V, and ice VI, respectively.

At each thermodynamic state point used in the corresponding enhanced-coexistence simulations, the liquid–ice II coexistence system was generated by combining a box of ice II, which was equilibrated with the DNN@MB-pol potential for 0.5 ns, and a box of liquid water (with a pair of opposite faces commensurate with the ice II box), which was equilibrated for 1.3 ns with the DNN@MB-pol potential, using the “modify-cell” functionality available in LAMMPS.

4.2 Enhanced coexistence simulations

Following best practices for ordinary direct-coexistence simulations,[61] we performed enhanced-coexistence simulations in the NP_xT ensemble (fluctuating box dimensions in the x-direction and constant area in the yz-directions) using the DNN@MB-pol potential. In these simulations, the box dimensions along the yz-directions were scaled to the average equilibrium values of the ice polymorph calculated at the corresponding thermodynamic condition of the enhanced-coexistence simulation.

We first carried out an initial sampling of 0.125 ns, 3 ns, 5 ns, 0.125 ns, and 0.125 ns for $\mathrm{I_h}$, ice II, ice III, ice V, and ice VI, respectively, in the $\mathrm{NP}_x\mathrm{T}$ ensemble with harmonic wall-bias potentials at n_{ice}^{low} and n_{ice}^{high} (see Table 2), confining the number ice molecules within these limits. This step allowed the coexistence system to equilibrate away from any initial biases due to the choice of the initial configuration, such as the coexistence being created with the TIP4P/Ice model. More importantly, this step also allowed for relaxation toward the thermodynamically favored outer ice range, which tends to speed up the convergence of the bias potential. It should be noted that the

Supplementary Table 2: **Systems setup for enhanced-coexistence simulations.** Total number of water molecules in the simulation box ($N_{\mathrm{H_2O}}$), length of the simulation box vectors, and range of ice-like molecules sampled in the enhanced-coexistence simulations.

	$N_{\mathrm{H_2O}}$	box sides (nm^3)	ice range
Liquid-$\mathrm{I_h}$	576	18x45x22	240-288
Liquid-II	864	31x23x35	432-486
Liquid-III	756	47x20x20	351-405
Liquid-V	672	19x47x18	336-392
Liquid-VI	1280	53x25x23	640-720

coexistence systems involving ice II and ice III were equilibrated for a longer time due to both the slow dynamics of liquid water in the supercooled regime and the convergence requiring a longer time than the other ice polymorphs. After this step, enhanced sampling was initiated in the multi-umbrella ensemble.[46] The environments for constructing the collective variables were obtained using the Environment Finder software.[41]

For all technical details, we refer the reader to the digital repository that includes all input files necessary to reproduce all of our simulations. The input files to reproduce all the enhanced-coexistence simulations are available on PLUMED-NEST as part of a collective effort to improve the transparency and reproducibility of enhanced-sampling molecular simulations.[62]

We determined the DNN@MB-pol melting points of each ice polymorph by carrying out enhanced-coexistence simulations at different temperatures along isobars. The variation in the number of ice-like molecules during the enhanced-coexistence simulations is shown in Supplementary Figures 9-13. The corresponding free-energy differences shown in Supplementary Figures 14-18 are, for the most part, linear, with some deviations from linearity found for ice VI. Similar deviations from linearity were also reported in Ref. 39. The differences in chemical potentials between liquid water and each ice polymorph shown in Supplementary Figures 19-23 are well-fitted by a linear line, indicating that all estimates are well converged.

4.3 Corrections to chemical potential differences

The MB-pol corrections to the DNN@MB-pol chemical potentials were calculated in two steps using thermodynamic perturbation theory. First, at each thermodynamic state point (T, P), we carried out NPT simulations for liquid water (2 ns) and the relevant ice polymorph (1 ns) using the DNN@MB-pol potential. Second, for all molecular configurations extracted every 25 fs from

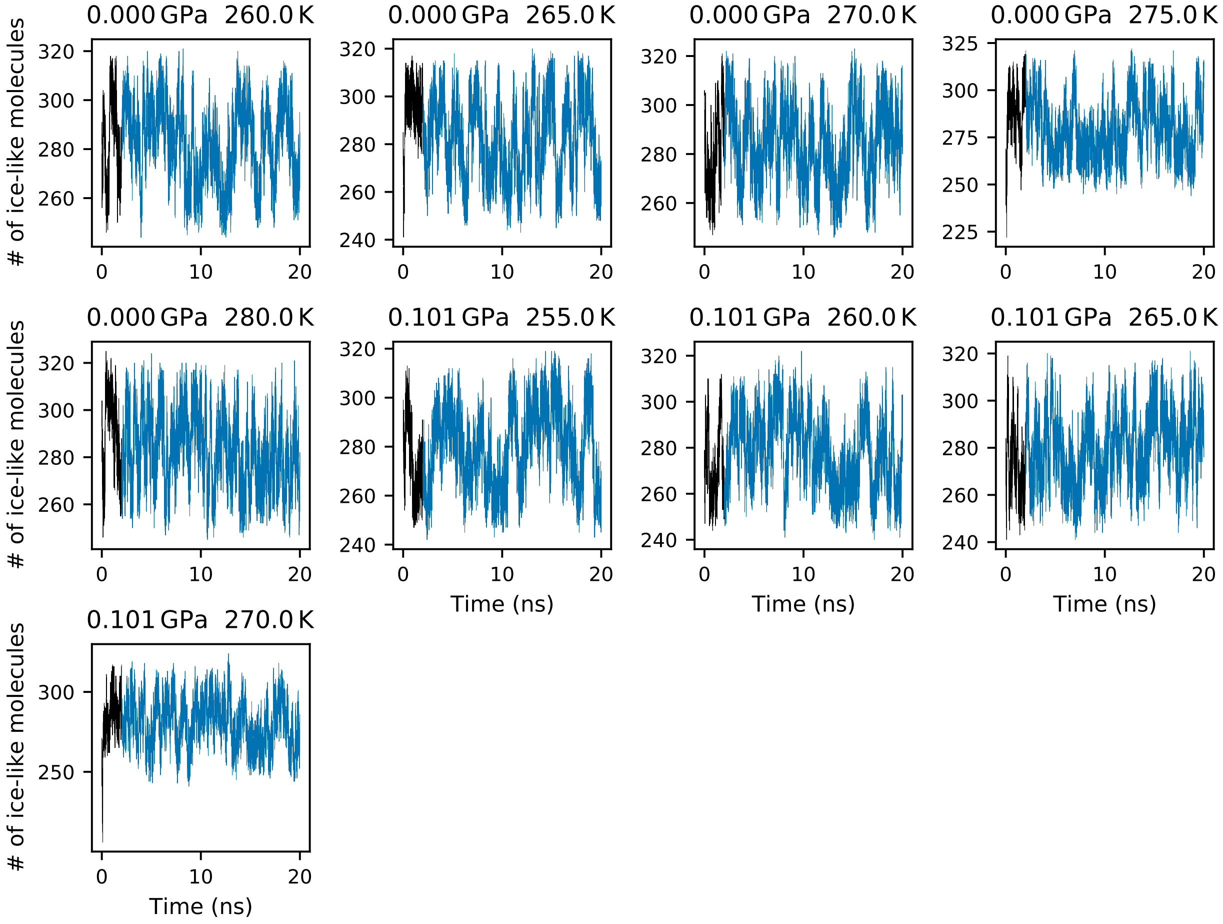

Supplementary Figure 9: **Liquid water–ice I_h coexistence.** Variation in the number of molecules classified as ice I_h along enhanced-coexistence simulations carried out with the DNN@MB-pol potential at different thermodynamic state points. In each enhanced-coexistence simulation, the equilibration time, shown in black, was disregarded in the melting point calculations because the bias potential changes rapidly during this time.

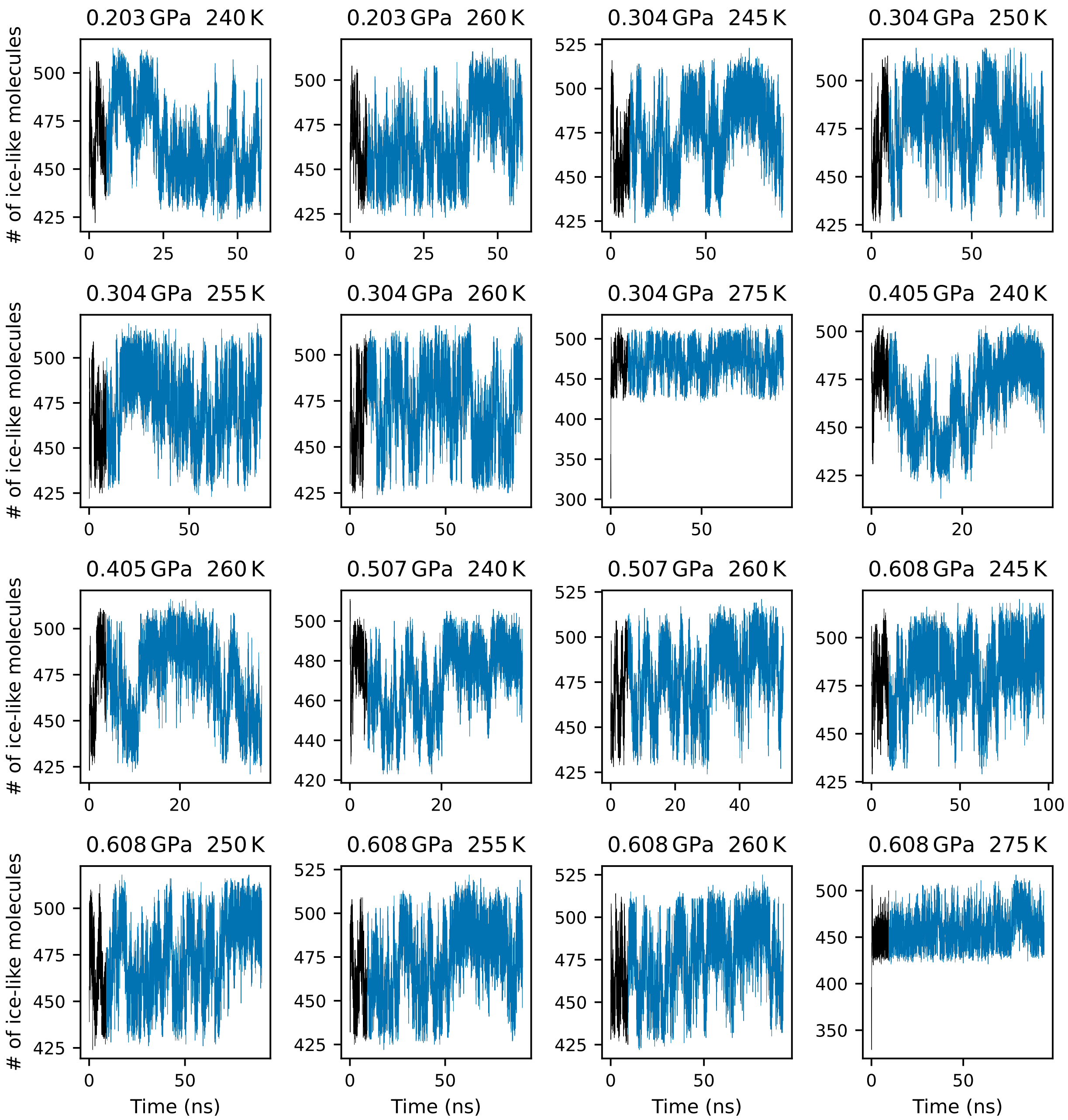

Supplementary Figure 10: **Liquid water–ice II coexistence.** Variation in the number of molecules classified as ice II along enhanced-coexistence simulations carried out with the DNN@MB-pol potential at different thermodynamic state points. In each enhanced-coexistence simulation, the equilibration time, shown in black, was disregarded in the melting point calculations because the bias potential changes rapidly during this time.

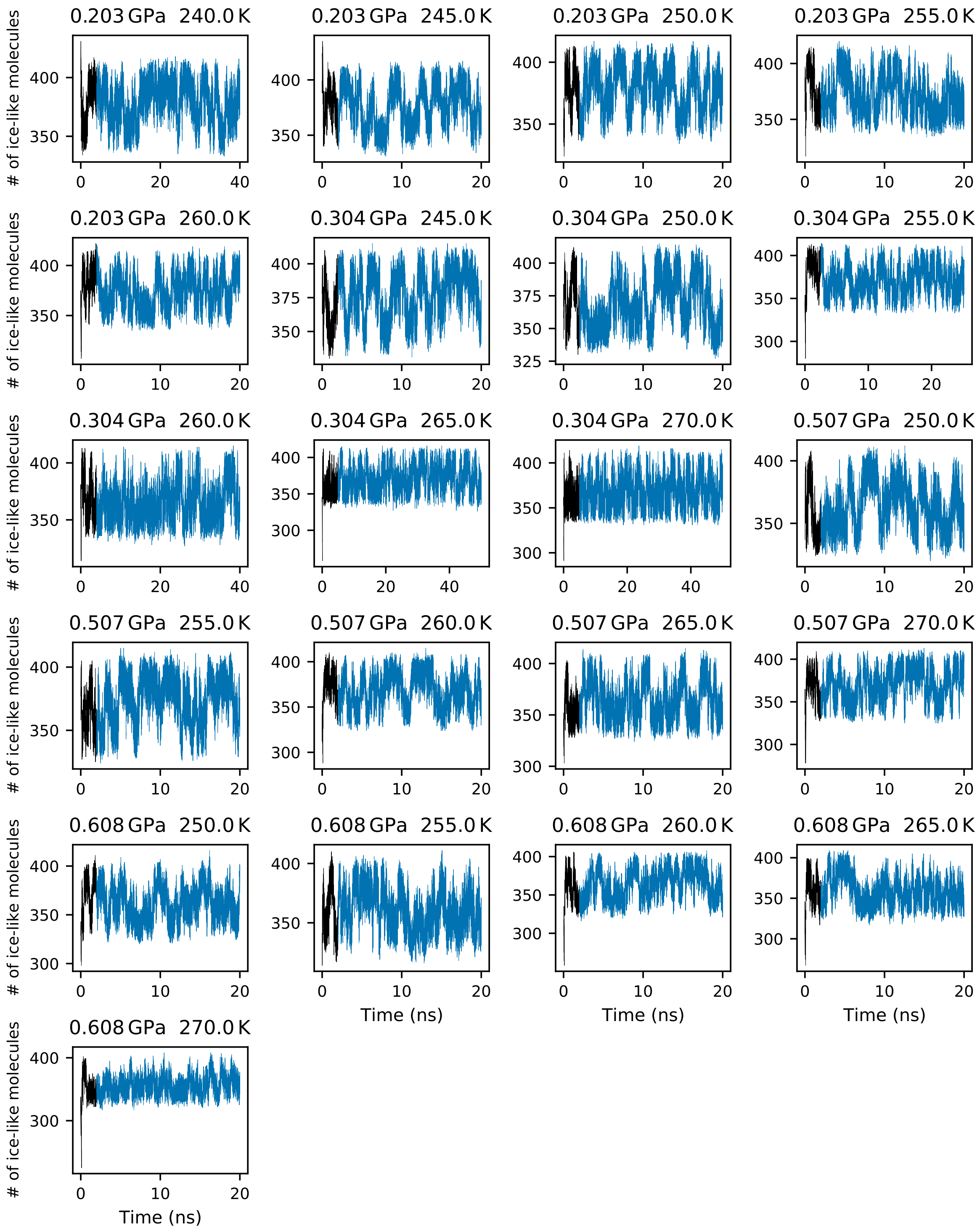

Supplementary Figure 11: **Liquid water–ice III coexistence.** Variation in the number of molecules classified as ice III along enhanced-coexistence simulations carried out with the DNN@MB-pol potential at different thermodynamic state points. In each enhanced-coexistence simulation, the equilibration time, shown in black, was disregarded in the melting point calculations because the bias potential changes rapidly during this time.

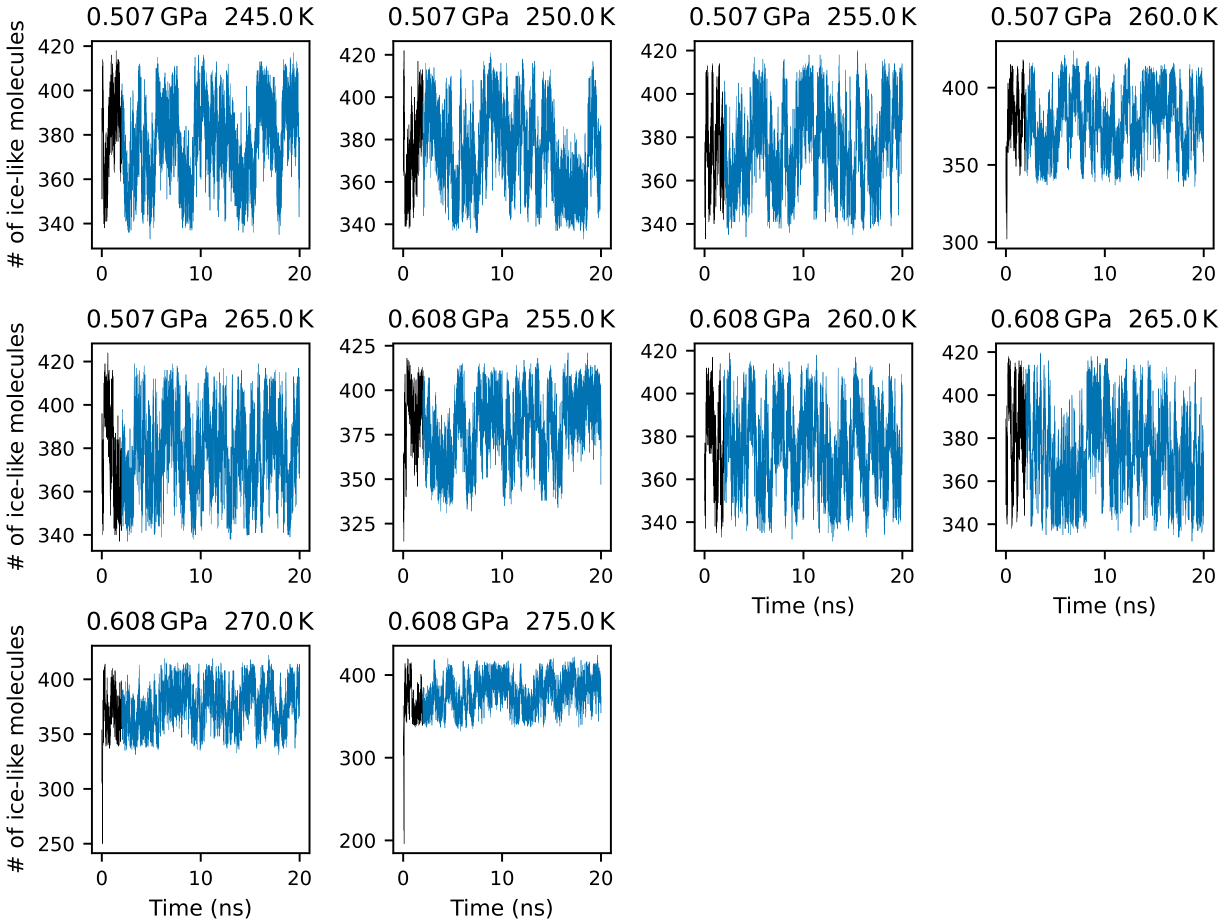

Supplementary Figure 12: **Liquid water–ice V coexistence.** Variation in the number of molecules classified as ice V along enhanced-coexistence simulations carried out with the DNN@MB-pol potential at different thermodynamic state points. In each enhanced-coexistence simulation, the equilibration time, shown in black, was disregarded in the melting point calculations because the bias potential changes rapidly during this time.

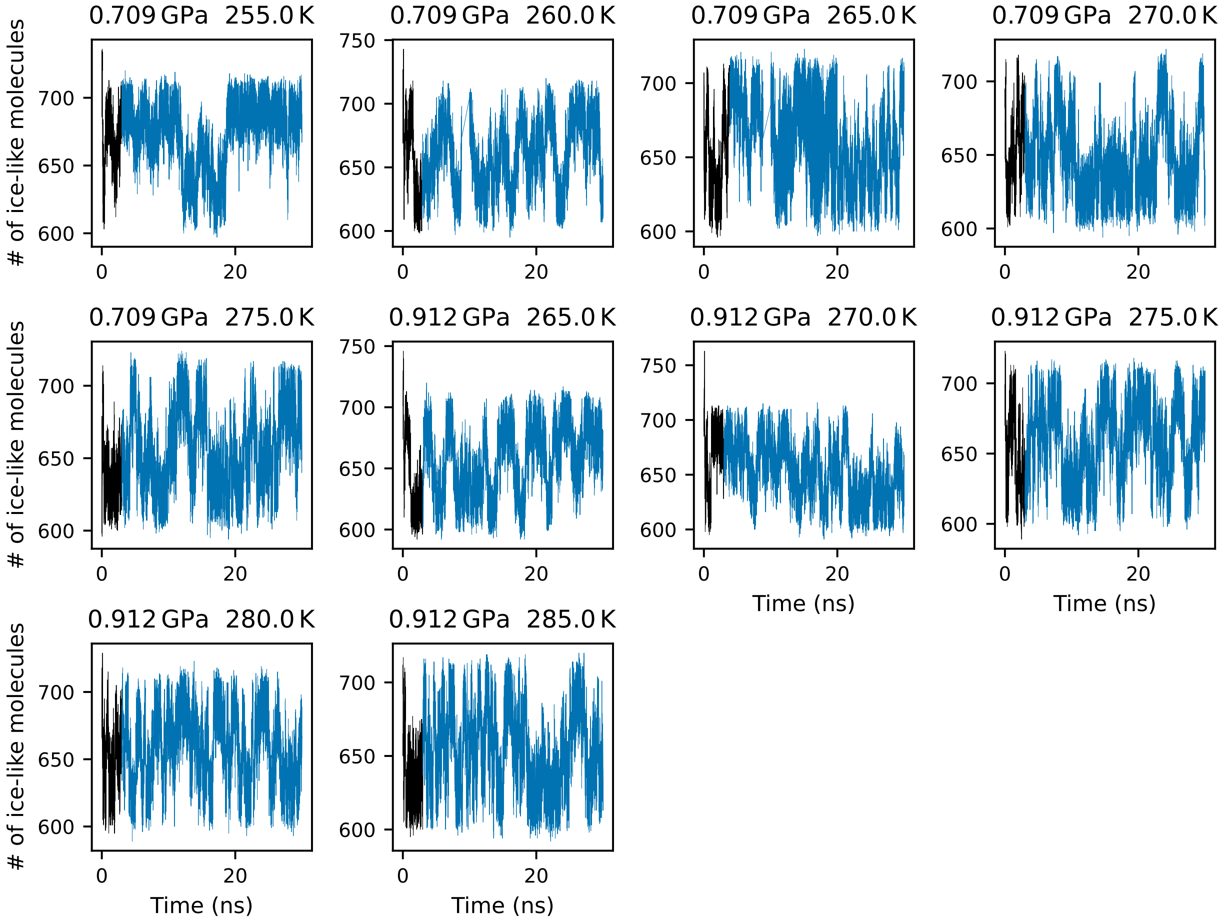

Supplementary Figure 13: **Liquid water–ice VI coexistence.** Variation in the number of molecules classified as ice VI along enhanced-coexistence simulations carried out with the DNN@MB-pol potential at different thermodynamic state points. In each enhanced-coexistence simulation, the equilibration time, shown in black, was disregarded in the melting point calculations because the bias potential changes rapidly during this time.

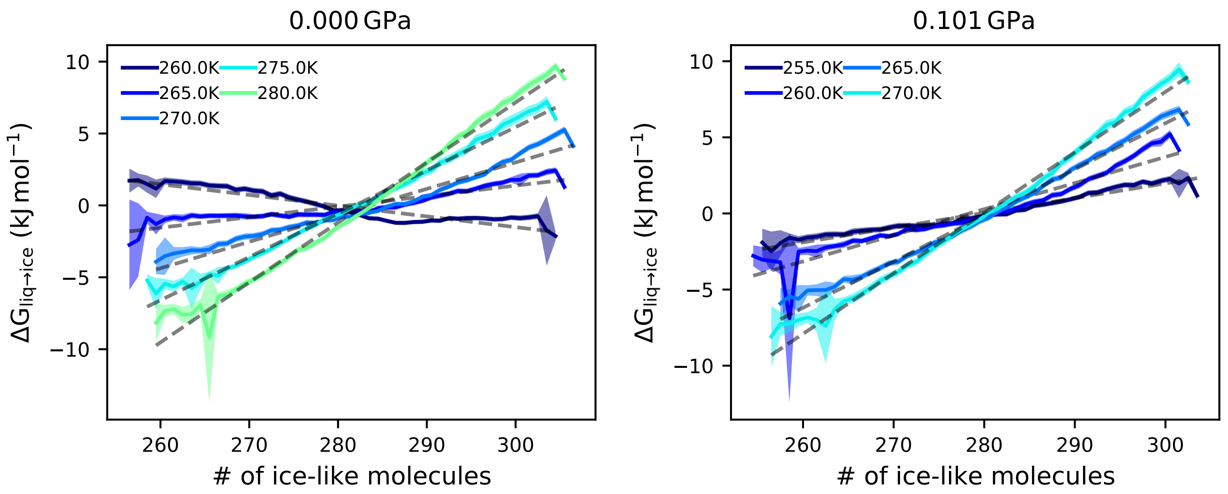

Supplementary Figure 14: **Liquid water–ice I$_h$ free-energy differences.** Free-energy differences between liquid water–ice I$_h$ ($\Delta G_{\text{liq}\rightarrow\text{ice}}$) calculated as a function of the number of ice I$_h$-like molecules from enhanced-coexistence simulations carried out with the DNN@MB-pol potential.

the DNN@MB-pol NPT simulations of each liquid water–ice polymorph pair, we calculated the corresponding MB-pol energies that were then used in thermodynamic perturbation calculations to determine the MB-pol corrections to the DNN@MB-pol chemical potentials according to Eq. 10.

The MB-pol corrections to the DNN@MB-pol chemical potentials calculated from thermodynamic perturbation theory are shown in Supplementary Figure 24. All corrections show low sensitivity to the thermodynamic conditions and are instead dependent on the specific ice polymorph (as also shown in Figure 3 of the main text). In general, elevating DNN@MB-pol to MB-pol stabilizes the ice polymorphs relative to liquid water, pushing the melting line toward higher temperatures. Interestingly, for ice II, there are minor corrections, which consequently decreases the stability of ice II relative to all other phases.

Quantum corrections to the classical MB-pol chemical potential differences were calculated by

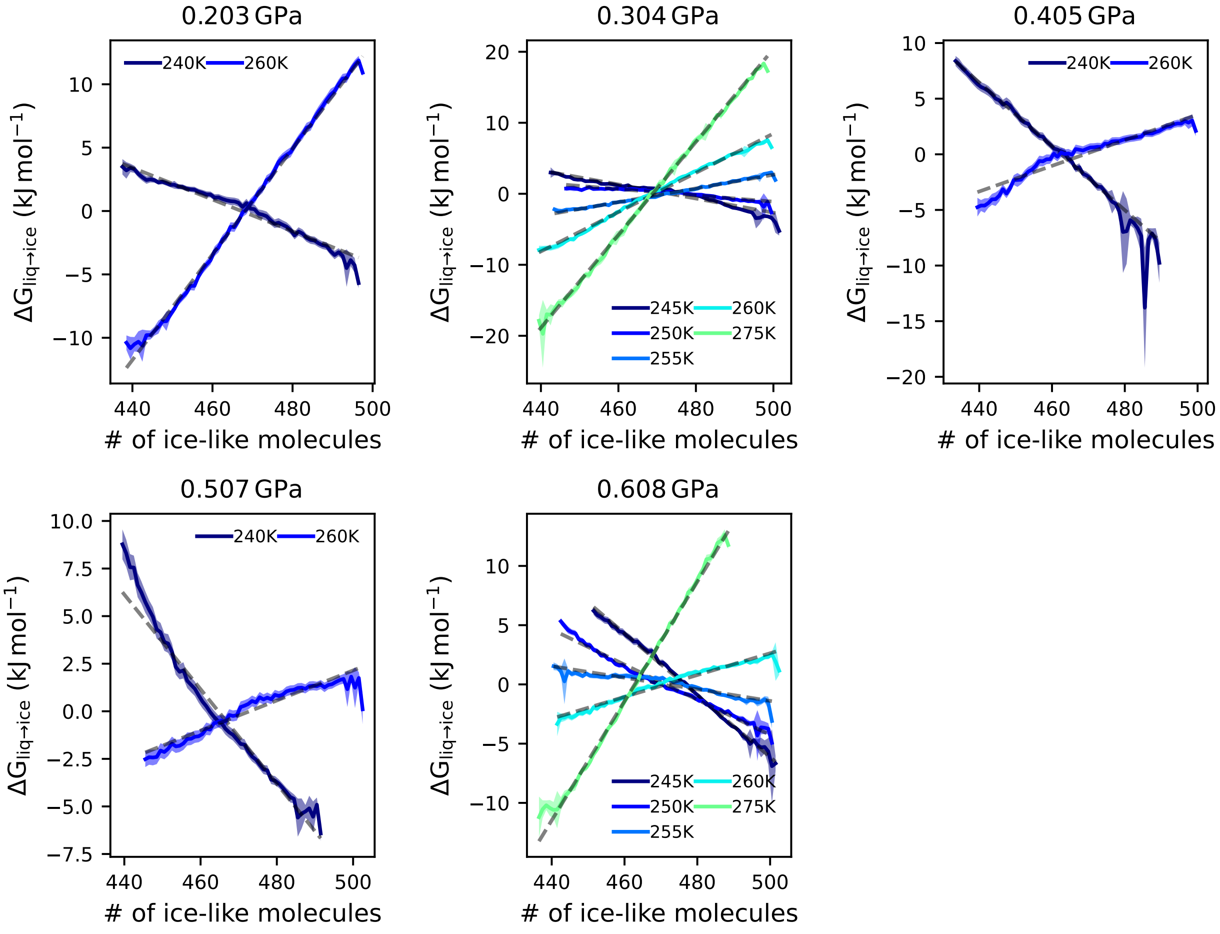

Supplementary Figure 15: **Liquid water–ice II free-energy differences.** Free-energy differences between liquid water–ice II calculated as a function of the number of ice II-like molecules from enhanced-coexistence simulations carried out with the DNN@MB-pol potential.

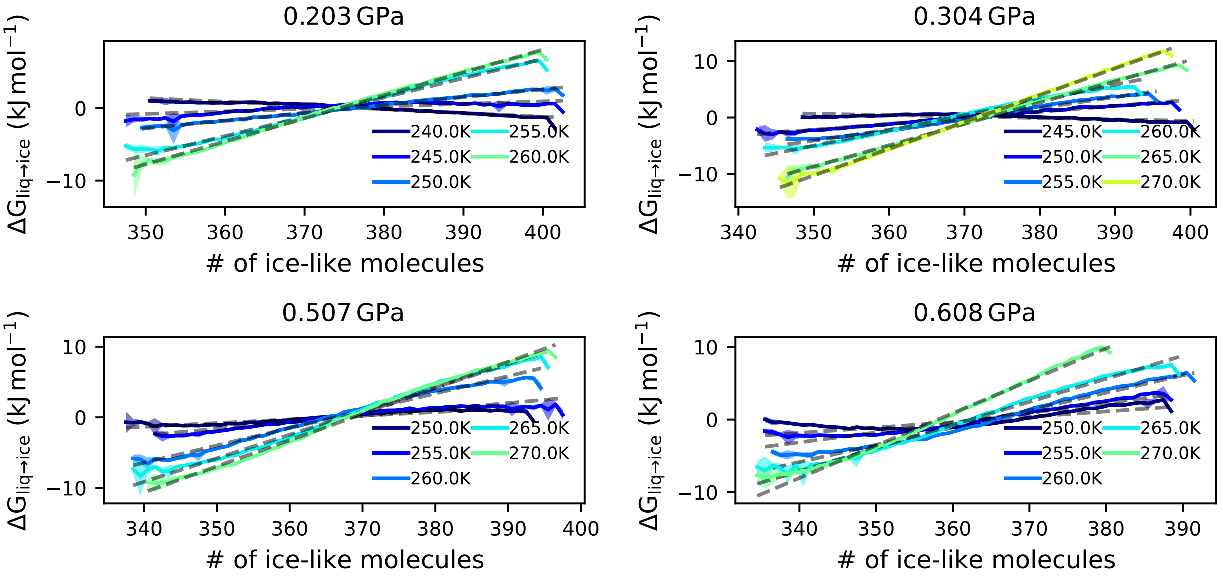

Supplementary Figure 16: **Liquid water–ice III free-energy differences.** Free-energy differences between liquid water–ice III ($\Delta G_{\text{liq}\to\text{ice}}$) calculated as a function of the number of ice III-like molecules from enhanced-coexistence simulations carried out with the DNN@MB-pol potential.

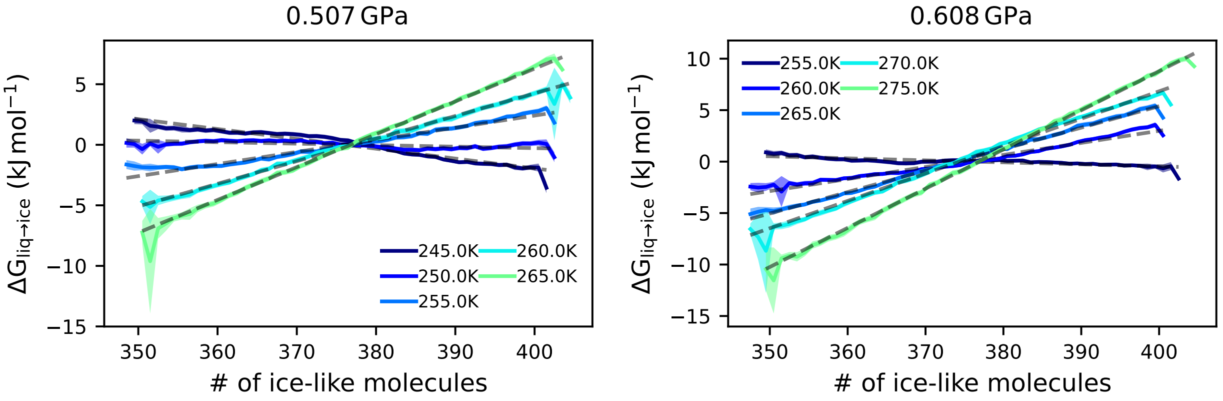

Supplementary Figure 17: **Liquid water–ice V free-energy differences.** Free-energy differences between liquid water–ice V ($\Delta G_{\text{liq}\to\text{ice}}$) calculated as a function of the number of ice V-like molecules from enhanced-coexistence simulations carried out with the DNN@MB-pol potential.

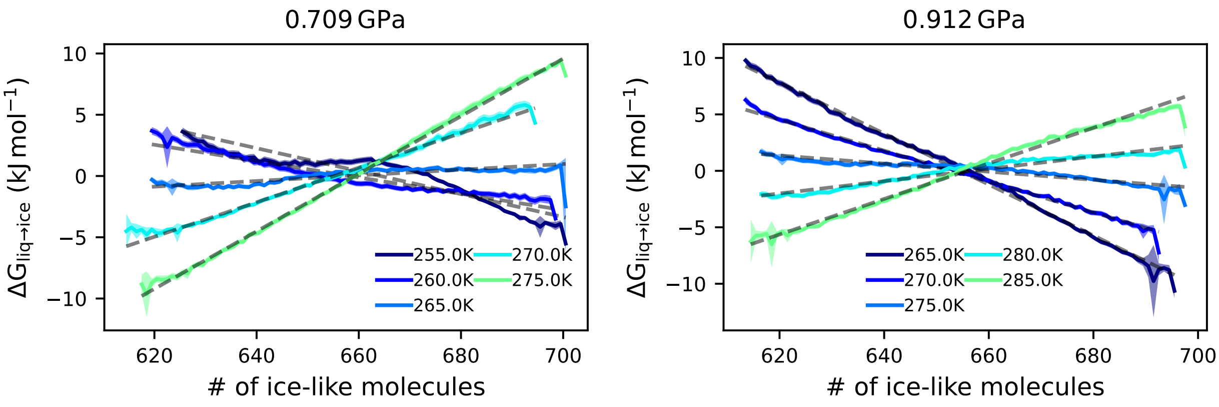

Supplementary Figure 18: **Liquid water–ice VI free-energy differences.** Free-energy differences between liquid water–ice VI ($\Delta G_{\mathrm{liq}\to\mathrm{ice}}$) calculated as a function of the number of ice VI-like molecules from enhanced-coexistence simulations carried out with the DNN@MB-pol potential.

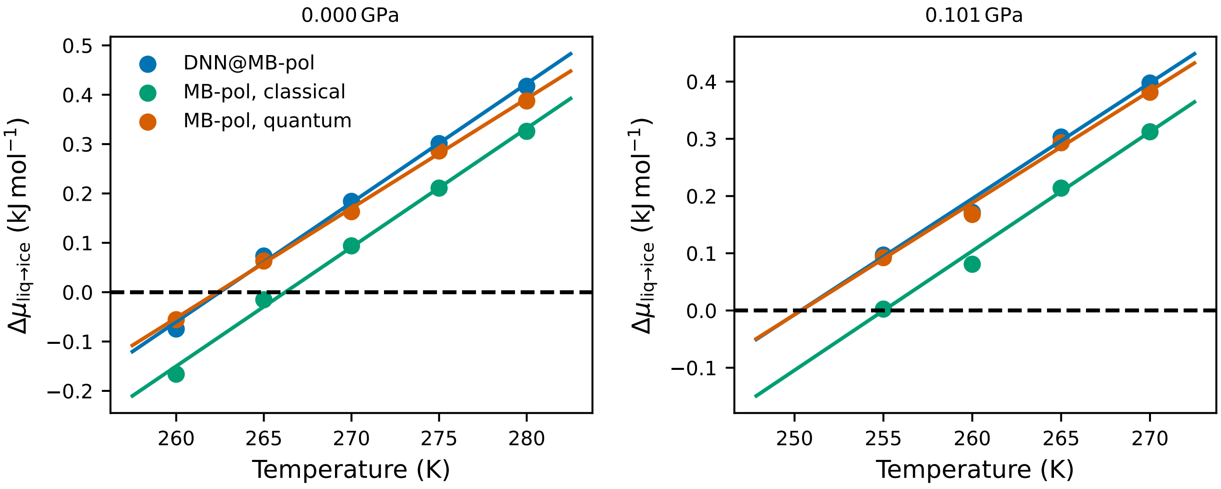

Supplementary Figure 19: **Liquid water–ice I$_\mathrm{h}$ chemical potential difference.** Temperature dependence of the chemical potential difference between liquid water and ice I$_\mathrm{h}$ ($\Delta\mu_{\mathrm{liq}\to\mathrm{ice}}$) determined from enhanced-coexistence simulations carried out with the DNN@MB-pol potential.

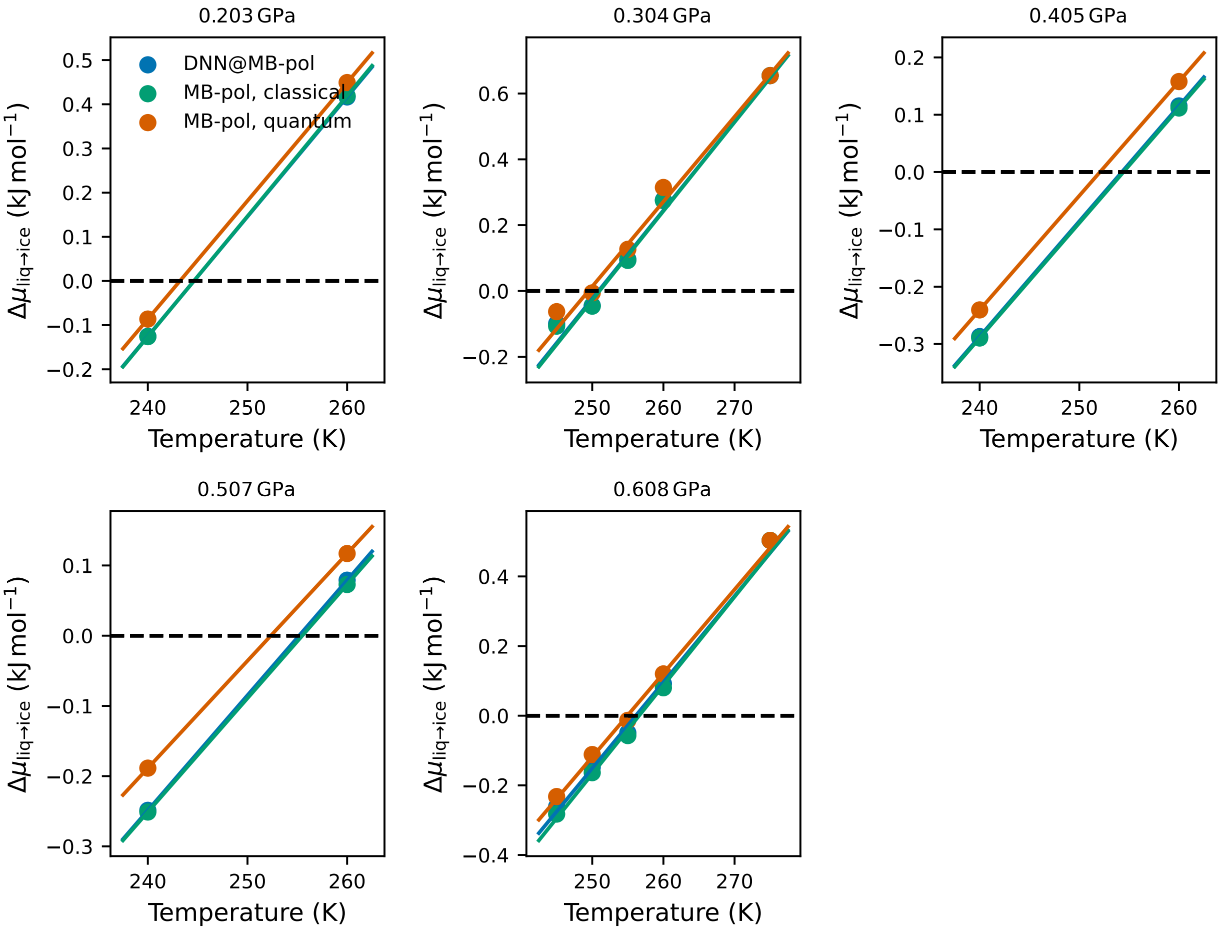

Supplementary Figure 20: **Liquid water–ice II chemical potential difference.** Temperature dependence of the chemical potential difference between liquid water and ice II ($\Delta\mu_{\mathrm{liq}\rightarrow\mathrm{ice}}$) determined from enhanced-coexistence simulations carried out with the DNN@MB-pol potential.

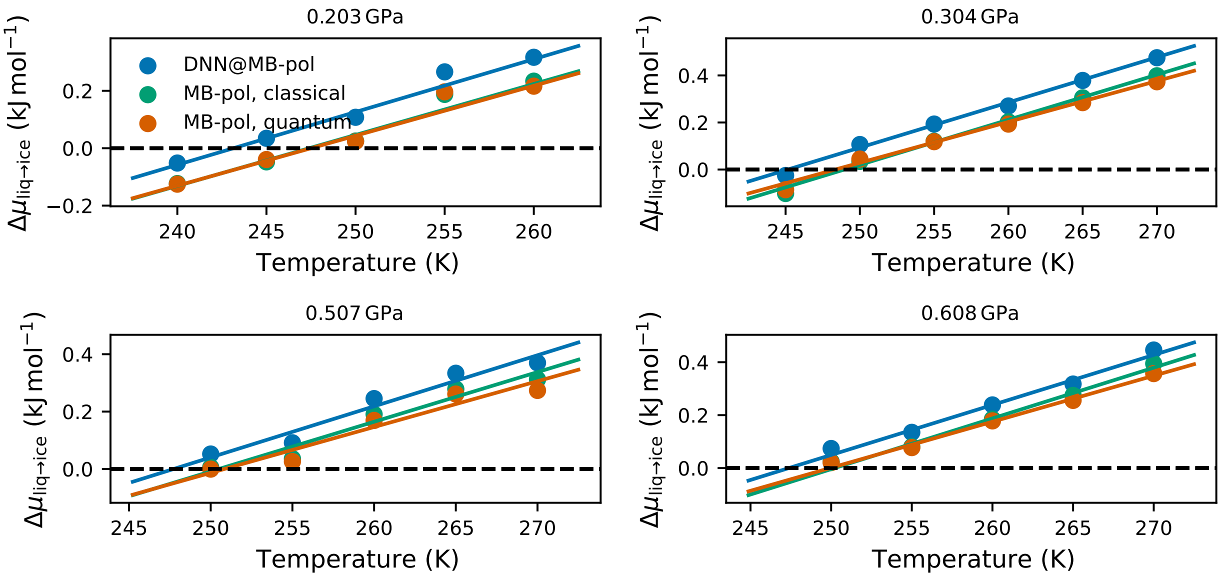

Supplementary Figure 21: **Liquid water–ice III chemical potential difference.** Temperature dependence of the chemical potential difference between liquid water and ice III ($\Delta\mu_{\mathrm{liq}\to\mathrm{ice}}$) determined from enhanced-coexistence simulations carried out with the DNN@MB-pol potential.

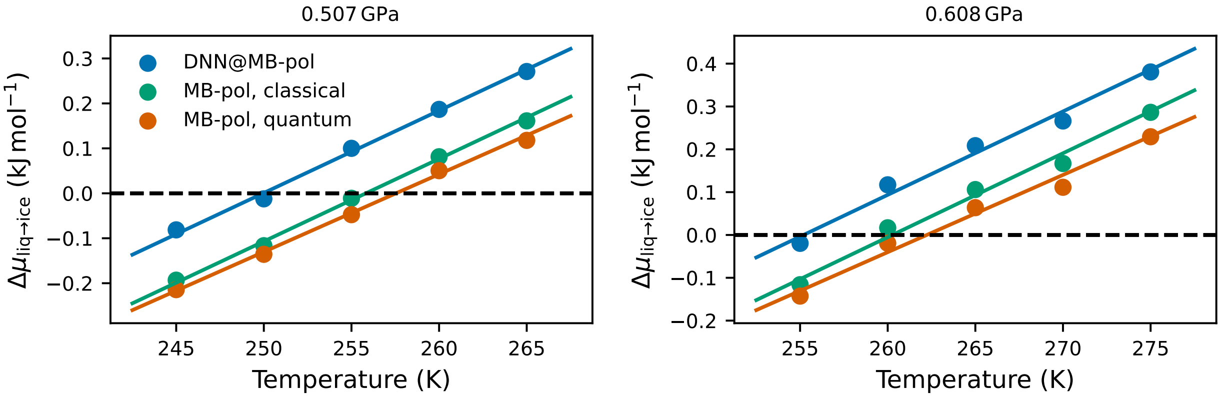

Supplementary Figure 22: **Liquid water–ice V chemical potential difference.** Temperature dependence of the chemical potential difference between liquid water and ice V ($\Delta\mu_{\mathrm{liq}\to\mathrm{ice}}$) determined from enhanced-coexistence simulations carried out with the DNN@MB-pol potential.

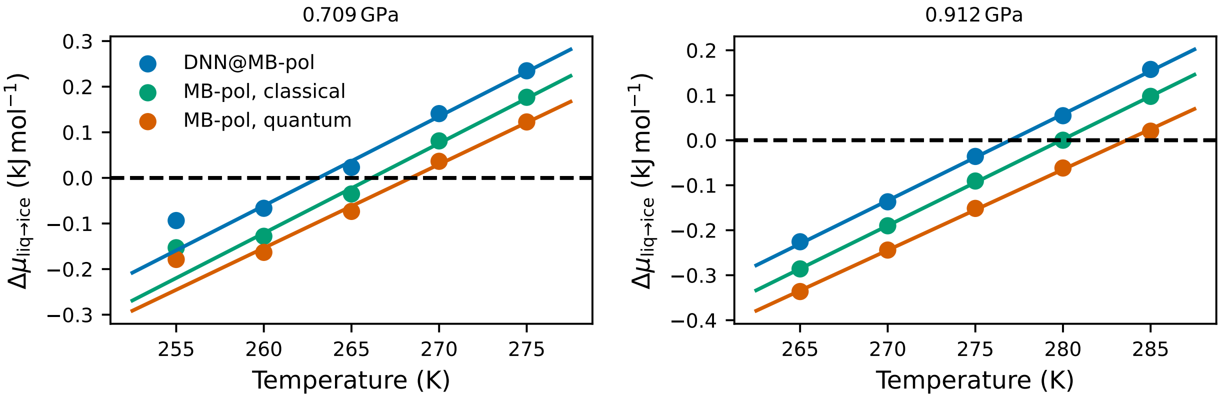

Supplementary Figure 23: **Liquid water–ice VI chemical potential difference.** Temperature dependence of the chemical potential difference between liquid water and ice VI ($\Delta\mu_{\mathrm{liq}\rightarrow\mathrm{ice}}$) determined from enhanced-coexistence simulations carried out with the DNN@MB-pol potential.

performing path-integral molecular dynamics (PIMD) simulations[54,55] using the same sets of configurations for liquid water and each ice polymorph used to determine the MB-pol classical corrections to the DNN@MB-pol classical chemical potential differences. Since the centroid kinetic energy is largely insensitive to the choice of water model,[63] due to computational cost considerations, we calculated the centroid kinetic energy with DNN@MB-pol, which enables more efficient PIMD simulations than MB-pol. The same strategy was adopted in previous studies where the quantum corrections to the chemical potential differences were calculated using neural network potentials instead of the reference DFT models.[48,52,64] The PIMD simulations were carried out using 32 beads and a time step of 0.25 fs. As in Ref. 52, the temperature was controlled by a PILE-G thermostat with a time constant of 10 fs for the global centroid thermostat,[65] and 100 fs for the stochastic barostat as detailed in Ref. 66. All PIMD simulations were carried out using i-PI2[67] interfaced with DeePMD-kit.[22]

The calculated quantum corrections to the MB-pol classical chemical potential differences are

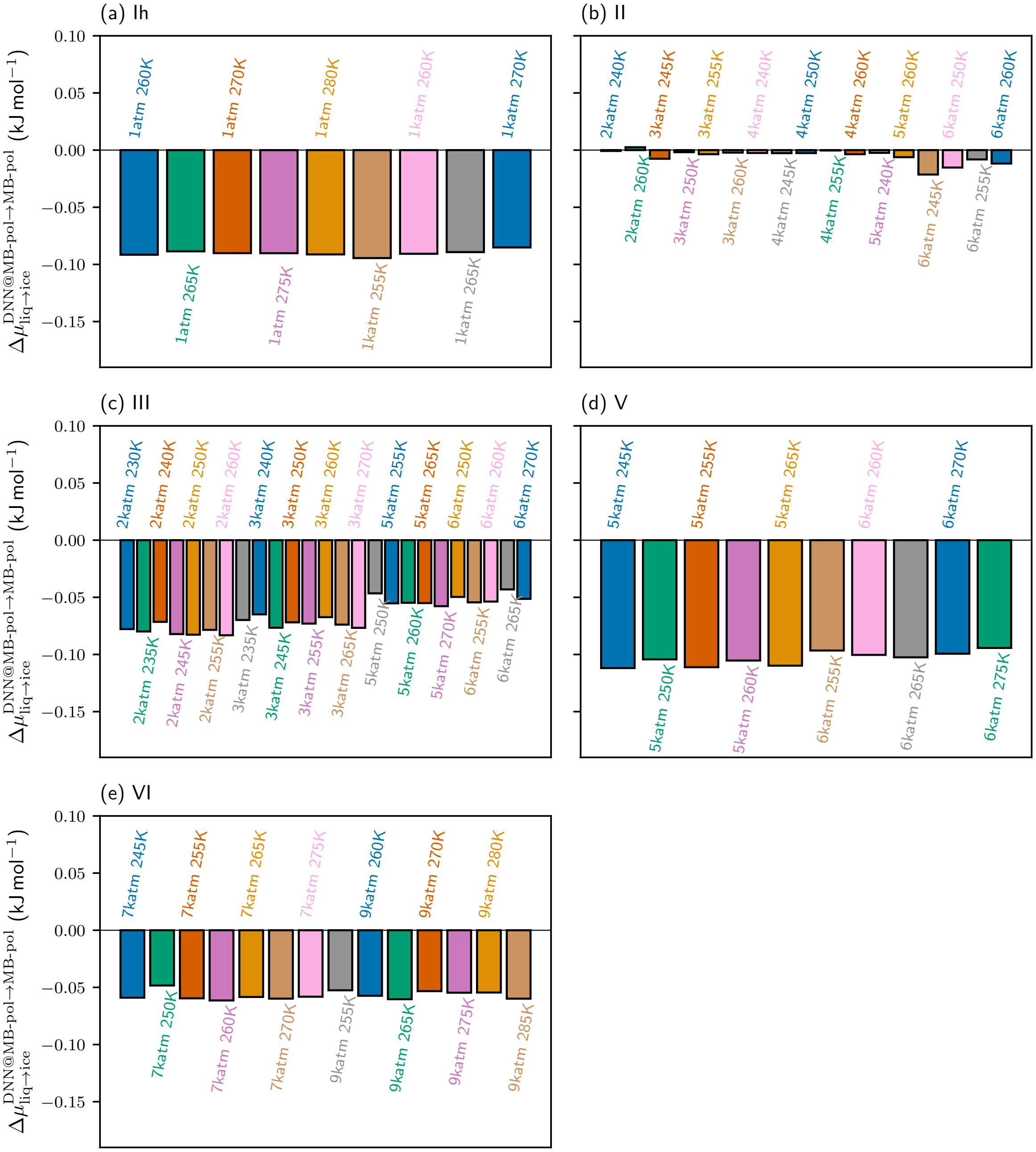

Supplementary Figure 24: **MB-pol corrections to the DNN@MB-pol chemical potentials.** MB-pol corrections to the DNN@MB-pol differences in chemical potentials between liquid water and each ice polymorph ($\Delta\mu_{\mathrm{liq}\rightarrow\mathrm{ice}}$). The MB-pol corrections were calculated using thermodynamic perturbation theory.

shown in Figures 25 and 26. Compared to the MB-pol classical corrections to the DNN@MB-pol classical chemical potential differences, the quantum corrections exhibit a larger variance. This is in part due to higher uncertainties associated with the corresponding thermodynamic integration calculations, which, due to computational cost considerations, were carried out using only five points to discretize the integration interval. However, as shown in Supplementary Figures 25 and 26, both the integrand and the centroid kinetic virial energy, along with the corresponding quantum corrections, exhibit clear trends as a function of the (T,P) thermodynamic state points, with the differences being primarily determined by the specific ice polymorph. In general terms, ice $\mathrm{I_h}$ and ice II are destabilized by nuclear quantum effects, while the high-pressure ice polymorphs, ice V and ice VI, are stabilized by nuclear quantum effects. Ice III is relatively unaffected by nuclear quantum effects.

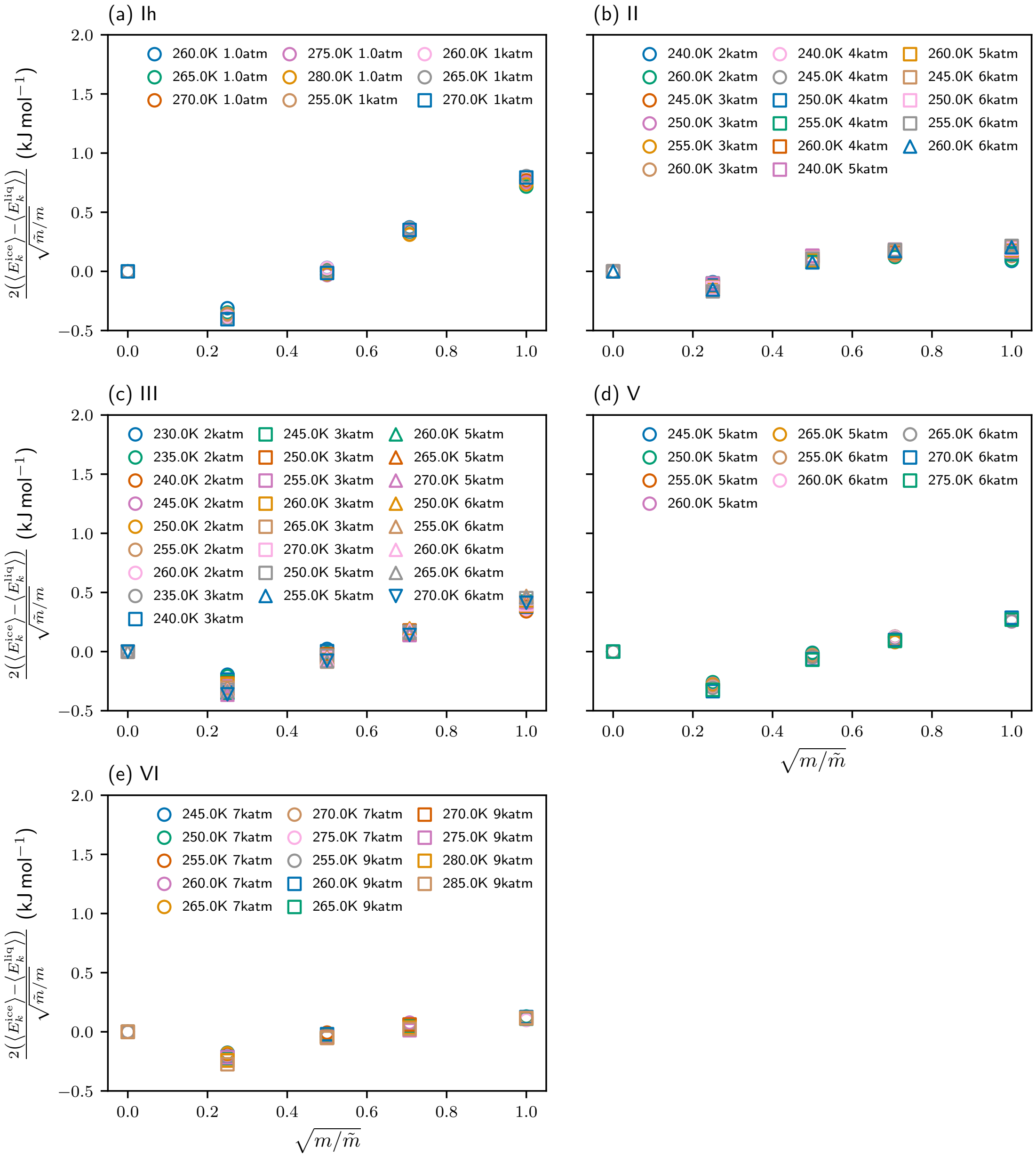

Supplementary Figure 25: **Nuclear quantum effects by thermodynamic integration.** The integrands used in thermodynamic integration by mass calculations to determine the quantum correction to the MB-pol classical chemical potential differences are shown as a function of the integration variable for all liquid water–ice polymorph coexistence equilibria. E_k is the centroid virial kinetic energy, while m and $\tilde{m}$ are the mass of the water molecule and the mass used during integration, respectively.

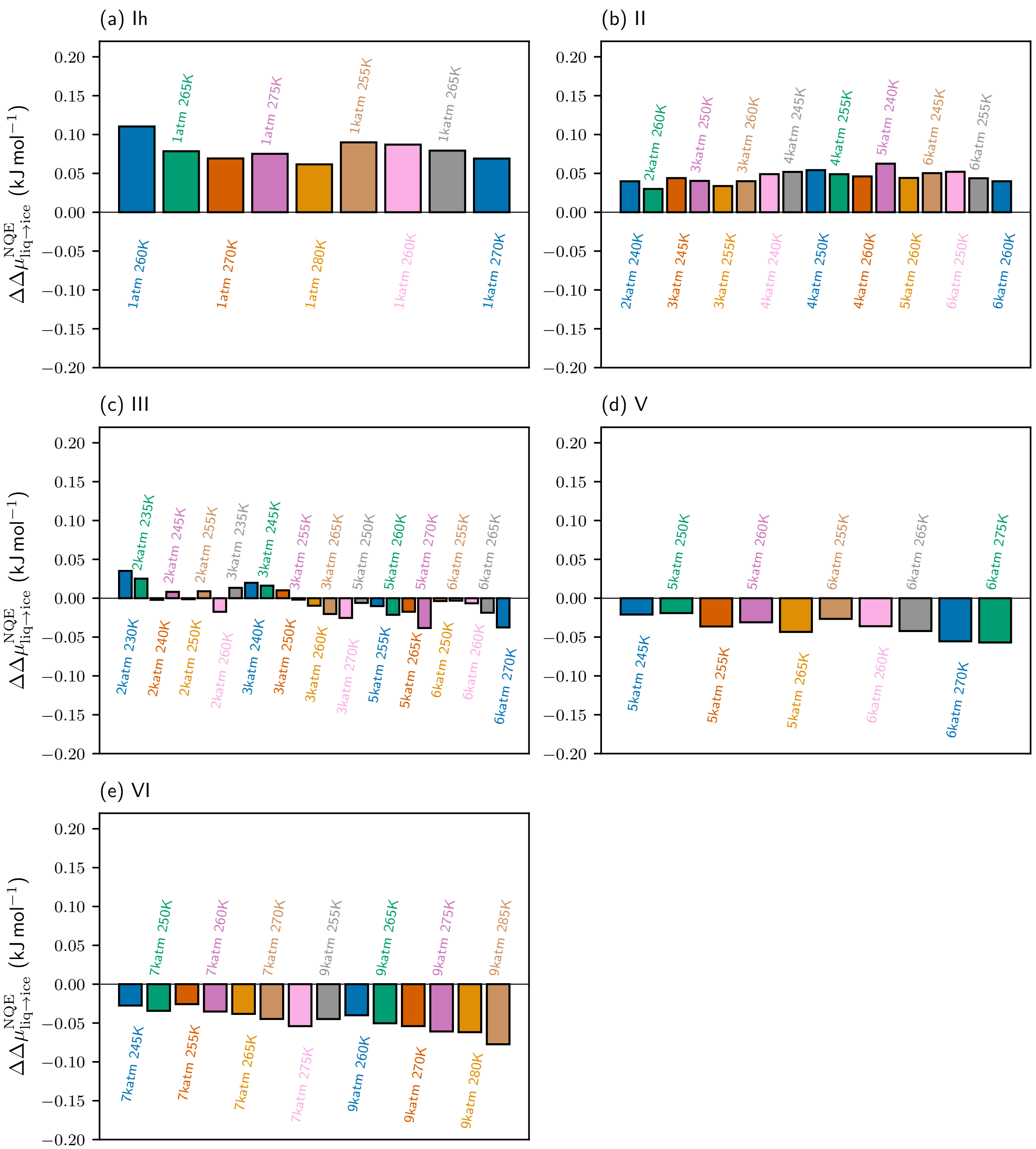

Supplementary Figure 26: **Quantum corrections to classical chemical potentials.** MB-pol quantum corrections to the MB-pol differences in chemical potentials between liquid water and each ice polymorph ($\Delta\mu_{\mathrm{liq}\to\mathrm{ice}}$). The quantum corrections were calculated using thermodynamic integration by mass.

4.4 Melting points

The melting points of the ice polymorphs at various pressures calculated from linear fits to the corresponding chemical potential differences with liquid water as described in Supplementary Note 2 are listed in Supplementary Tables 3-6. The accuracy of the present calculations can be assessed through comparisons with analogous results reported in the literature for the MB-pol potential (Table 3). The melting point of ice I_h calculated at ambient pressure from enhanced-coexistence simulations is 266.2 K, which is 2.7 K higher than the value reported in Ref. 7 which was obtained from direct-coexistence simulations. In Ref. 39, a difference of $\sim$1 K was found between estimates of the melting point of ice I_h obtained from enhanced-coexistence and direct-coexistence simulations carried out with the TIP4P/Ice model. Given typical errors of $\sim$2 K associated with direct coexistence-simulations,[68,69] which are mainly due to finite-size and interface artifacts,[70,71] the difference between the enhanced-coexistence and direct-coexistence melting points of ice I_h simulated with the MB-pol potential is consistent with analogous differences reported in the literature for other water models.

Supplementary Table 3: **Classical and quantum melting point (T_m) and heat of fusion for ice I_h (H_{fus}) calculated at 1 atm with the DNN@MB-pol and MB-pol potentials.**

Method	T_m/K	H_{fus}/kJ mol^{-1}
H_2O, experiment	273.15	6.01
D_2O, experiment	276.95	6.22
DNN@MB-pol (this work)	262.5	6.33
Classical MB-pol (this work)	266.2	6.42
Quantum MB-pol (this work)	262.3	5.83
Classical MB-pol Ref. 7	263.5	N/A

Supplementary Table 4: **Classical melting points of the ice polymorphs calculated with the DNN@MB-pol potential.**

	P/GPa	T/K
I_h-Liq	0.0001	262.5
I_h-Liq	0.1013	250.3
II-Liq	0.2026	244.6
II-Liq	0.3040	250.9
II-Liq	0.4053	254.3
II-Liq	0.5066	255.2
II-Liq	0.6079	256.1
III-Liq	0.2026	243.2
III-Liq	0.3040	245.2
III-Liq	0.5066	247.7
III-Liq	0.6079	247.4
V-Liq	0.5066	250.0
V-Liq	0.6079	255.2
VI-Liq	0.7093	263.1
VI-Liq	0.9119	277.0

Supplementary Table 5: **Classical melting points of the ice polymorphs calculated with the MB-pol potential.**

	P/GPa	T/K
I_h-Liq	0.0001	266.2
I_h-Liq	0.1013	255.0
II-Liq	0.2026	244.6
II-Liq	0.3040	251.0
II-Liq	0.4053	254.4
II-Liq	0.5066	255.5
II-Liq	0.6079	256.6
III-Liq	0.2026	247.4
III-Liq	0.3040	249.0
III-Liq	0.5066	250.5
III-Liq	0.6079	250.2
V-Liq	0.5066	255.8
V-Liq	0.6079	260.3
VI-Liq	0.7093	266.1
VI-Liq	0.9119	279.9

Supplementary Table 6: Quantum melting points of the ice polymorphs calculated with the MB-pol potential.

	P/GPa	T/K
I_h-Liq	0.0001	262.4
I_h-Liq	0.1013	250.4
II-Liq	0.2026	243.2
II-Liq	0.3040	249.5
II-Liq	0.4053	252.1
II-Liq	0.5066	252.3
II-Liq	0.6079	254.9
III-Liq	0.2026	247.5
III-Liq	0.3040	248.3
III-Liq	0.5066	250.8
III-Liq	0.6079	249.9
V-Liq	0.5066	257.5
V-Liq	0.6079	262.2
VI-Liq	0.7093	268.4
VI-Liq	0.9119	283.6

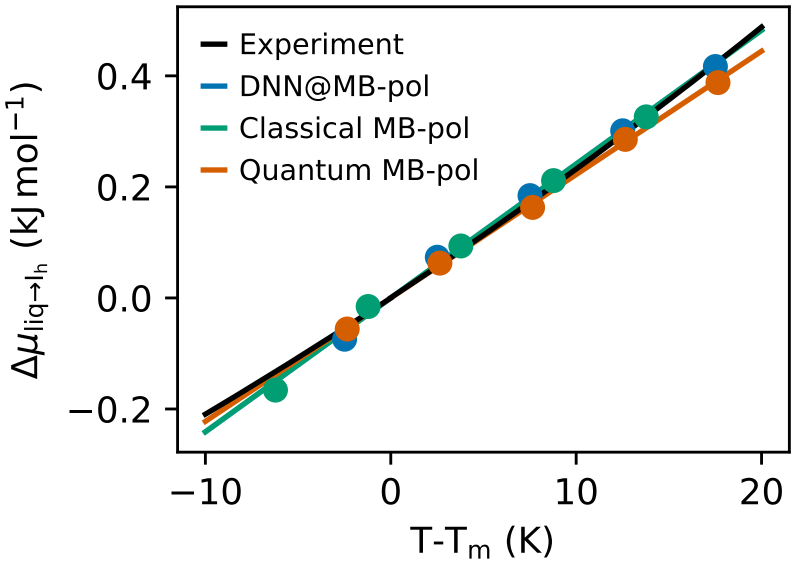

Supplementary Figure 27: Chemical potential for Ice I_h. Estimated chemical potentials of ice Ih compared with experiments as a function of temperature relative to the melting point. The experimental curve was calculated from the heat capacities reported in Ref. 72.

Besides the melting points, the chemical potential differences determined from the enhanced-coexistence simulations provide valuable insights into the thermodynamics of ice I_h melting. Supplementary Figure 27 shows the chemical potential difference between liquid water and ice I_h calculated with MB-pol as a function of temperature relative to the corresponding melting point (T_m). Upon accounting for the differences in melting points, MB-pol provides remarkable agreement with the experimental chemical potential difference, which is reflected in highly accurate estimates for the heats of fusion of both H_2O and D_2O ice I_h (see main text).

4.5 Triple points and phase diagrams

From the triple points of the liquid–ice coexistence lines, we iteratively performed additional Gibbs-Duhem integration calculations to determine all triple points, which are listed in Supplementary Tables 7-9. These additional Gibbs-Duhem integration calculations also allowed us to determine the entire phase diagrams of water at the classical level with the DNN@MB-pol and MB-pol potentials, and at the quantum-mechanical level with the MB-pol potential, which are shown in Figure 2 of the main text.

Specifically, a series of NPT simulations were carried out to perform the Gibbs-Duhem integration calculations necessary to trace all coexistence lines. For each thermodynamic state point, the NPT simulations were carried out until the estimated relative error on the volume per molecule was 0.05%, and the first 20% of each NPT trajectory was disregarded as equilibration time where the bias potential typically changes rapidly. To reduce the required equilibration time, for each integration point, we used the final configuration of the previous integration step as the starting

configuration. For steep coexistence lines in the temperature and pressure plane (i.e., liquid–ice II, liquid–ice III, liquid–ice V, liquid–ice VI, and ice II–ice III), the integration was carried out over the pressure using an integration step of 0.0253 GPa. For near-flat coexistence lines in the temperature and pressure plane (i.e., liquid–ice I_h, ice I_h–ice III, ice I_h–ice II, ice II–ice V, ice II–ice VI, ice III–ice V, and ice V–ice VI), the integration was instead carried out over the temperature, with an integration step of 5 K. To draw the coexistence lines between the liquid and each ice polymorph, we used the average of the Gibbs-Duhem lines calculated from each of the melting points. For the liquid–ice III coexistence, we used a quadratic polynomial fitted to the four melting points. To ensure sufficient sampling, which can require several tens of nanoseconds for supercooled water, we carried out the Gibbs-Duhem simulations with the DNN@MB-pol potential. The validity of approximating Gibbs-Duhem integration calculations for MB-pol, at both classical and quantum levels, with analogous calculations carried out with DNN@MB-pol is demonstrated empirically in Figure 1 of the main text.

Supplementary Table 7: **Classical triple points calculated with the DNN@MB-pol potential.**

	P/GPa	T/K
Liq-III-V	0.4706	247.4
Liq-I_h-II	0.1646	243.3
Liq-V-VI	0.6422	257.0
Liq-II-V	0.6325	256.6
Liq-II-VI	0.6375	256.6

Supplementary Table 8: **Classical triple points calculated with the MB-pol potential.**

	P/GPa	T/K
Liq-III-V	0.4326	250.2
Liq-I_h-II	0.1848	244.8
Liq-I_h-III	0.1714	246.7
Liq-V-VI	0.7030	265.2
Liq-II-V	0.5140	255.6
Liq-II-VI	0.6057	256.6
Liq-II-III	0.2506	248.2
I_h-II-III	0.1747	242.7
II-V-VI	0.6664	227.1

Supplementary Table 9: **Quantum triple points calculated with the MB-pol.**

	P/GPa	T/K
Liq-III-V	0.4035	249.9
Liq-I_h-II	0.1731	242.1
Liq-I_h-III	0.1437	246.0
Liq-V-VI	0.6748	265.8
Liq-II-V	0.4339	252.2
Liq-II-VI	0.5509	254.1
Liq-II-III	0.3239	249.1
I_h-II-III	0.1519	237.2
II-V-VI	0.6323	216.5

Supplementary Note 5: Einstein Molecule method & stability of ice III

For proton-disordered ice polymorphs, the Einstein Molecule method relies on Pauling's expression for the residual entropy of a crystal to determine the entropic contributions to the free-energy differences between the liquid and the given proton-disordered ice polymorph. As discussed in Ref. 73, this approach can straightforwardly be applied to ice polymorphs that present total proton disorder by simply calculating Pauling's entropy contributions for a "representative" configuration. For ice polymorphs with partial proton disorder, however, the selection of a "representative" configuration is nontrivial since the energy of different "representative" configurations varies significantly, which leads to significantly different free-energy values. In principle, the correct "representantive" configuration is the configuration that minimizes the associated free energy. In practice, identifying the correct "representantive" configuration for partially proton-disordered ice polymorphs is a daunting task to accomplish because determining the minimum free-energy configuration requires extremely long simulations due to the extremely slow transition from one configuration to another. This implies that all calculations of the phase diagram of water performed with the Einstein Molecule method are not able to correctly account for entropic contributions to the free energy of partially proton-disordered ice polymorphs.

In contrast, by explicitly simulating the crystallization process, direct-coexistence and enhanced-coexistence simulations do not rely on any approximations for the entropic contributions associated with proton disorder. This allows for correctly determining the free-energy difference between liquid water and a given ice polymorph, independently of the extent of proton disorder present in the

ice polymorph (i.e., direct-coexistence and enhanced-coexistence simulations inherently sample the relevant regions of the underlying multidimensional free-energy landscape).

The TIP4P/2005[73,74] and TIP4P/Ice[39,75] phase diagrams, calculated using both the Einstein Molecule method and direct-coexistence or enhanced-coexistence simulations, are shown in Figure 28. The

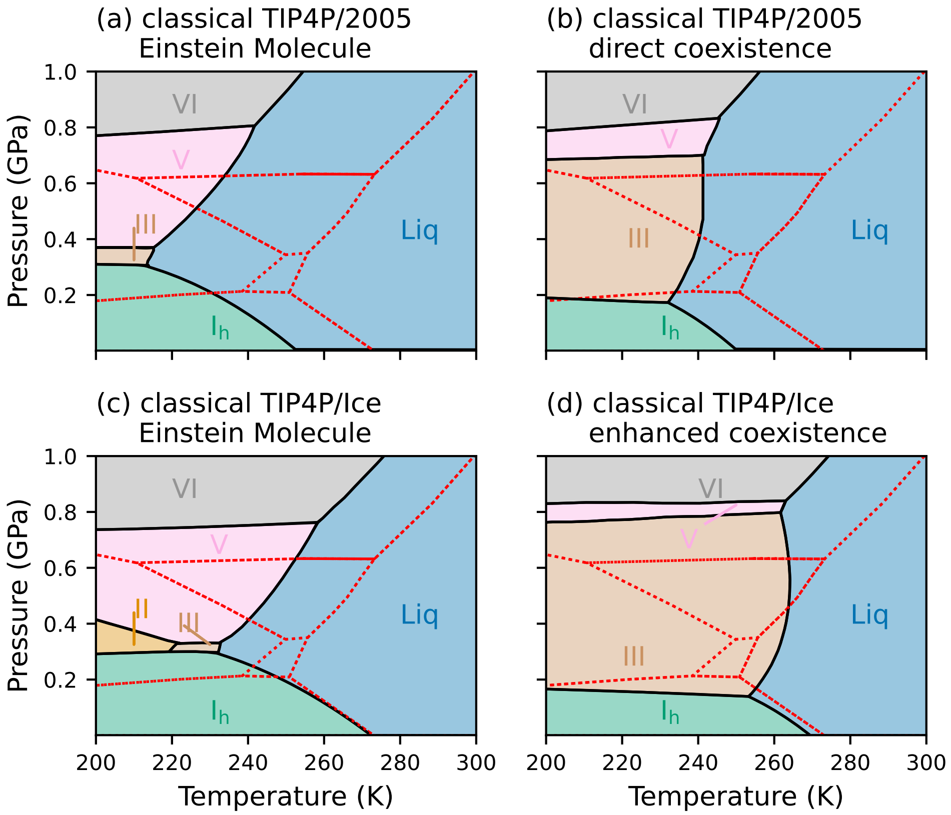

Supplementary Figure 28: **Comparison of phase diagrams from the TIP4P water model family.** **a)** Classical phase diagram of TIP4P/2005 from Ref. 74 calculated using the Einstein Molecule method. **b)** Classical phase diagram of TIP4P/2005 from Ref. 73 calculated using direct-coexistence simulations. **c)** Classical phase diagram of TIP4P/Ice from Ref. 75 calculated using the Einstein Molecule method. **d)** Classical phase diagram of TIP4P/Ice from Ref. 39 calculated using direct-coexistence simulations. The phase diagrams that are shown in **a)**, **c)**, and **d)** were digitized from the original references. The experimental phase diagram[33] is shown in each panel using a dotted red line.

comparisons clearly demonstrate that the Einstein Molecule method largely underestimates the region of stability for ice III, which thus results in an incorrect representation of the actual phase diagrams for both TIP4P/2005 and TIP4P/ice.

To date, the TIP4PQ/2005 phase diagram has only been calculated at the quantum level using the Einstein Molecule method.[51] Since Figure 28 shows that the Einstein Molecule method underestimates the melting points of ice III, we determined the correct melting points of ice III for the TIP4PQ/2005 model by performing multiple direct-coexistence simulations using the same coexistence setup as reported in Ref. 73. Supplementary Figure 29 shows the variation of the potential energy calculated in liquid–ice III direct-coexistence simulations performed at 0.4 GPa and different temperatures. While there is some stochasticity involved in direct-coexistence simulations, the time evolution clearly indicates a melting point in the vicinity of ~280-285 K. In comparison,

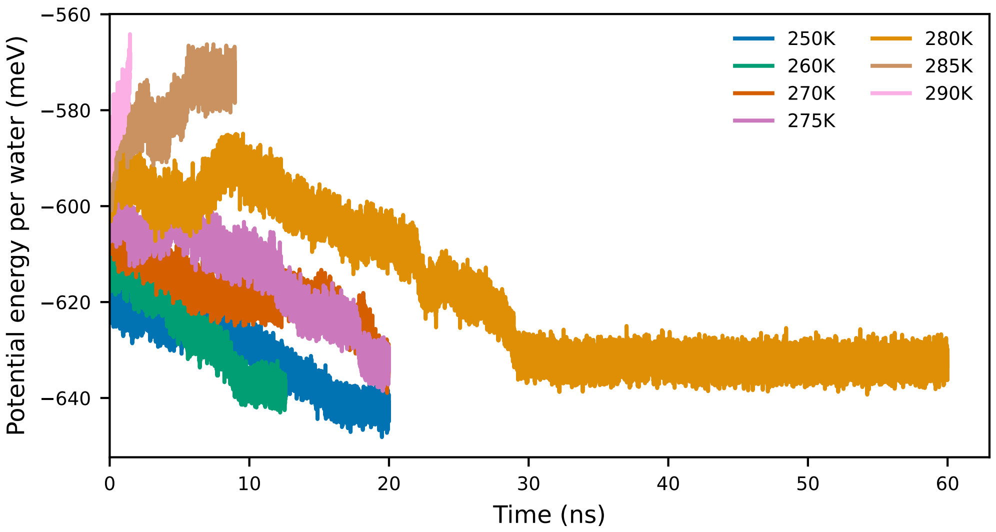

Supplementary Figure 29: **Direct-coexistence simulations with TIP4PQ/2005.** Variation of the potential energy per water molecule along liquid water–ice III direct-coexistence simulations carried out with the TIP4PQ/2005 model.

the Einstein Molecule method used in Ref. 51 predicts a melting point of ∼256 K at the same pressure, which is ∼25 K lower than the value obtained from our direct-coexistence simulations. This difference is consistent with a difference of ∼25 K between the melting points calculated with TIP4P/2005 using the Einstein Molecule method and direct-coexistence simulations. [73] As a consequence, the actual TIP4PQ/2005 phase diagram is expected to display a significantly larger area of stability for ice III than that originally reported in Ref. 51. For reference, a comparison between the original phase diagram of water calculated at the quantum level with the TIP4PQ/2005 model in Ref. 51 and the corresponding MB-pol phase diagram is shown in Supplementary Figure 30.

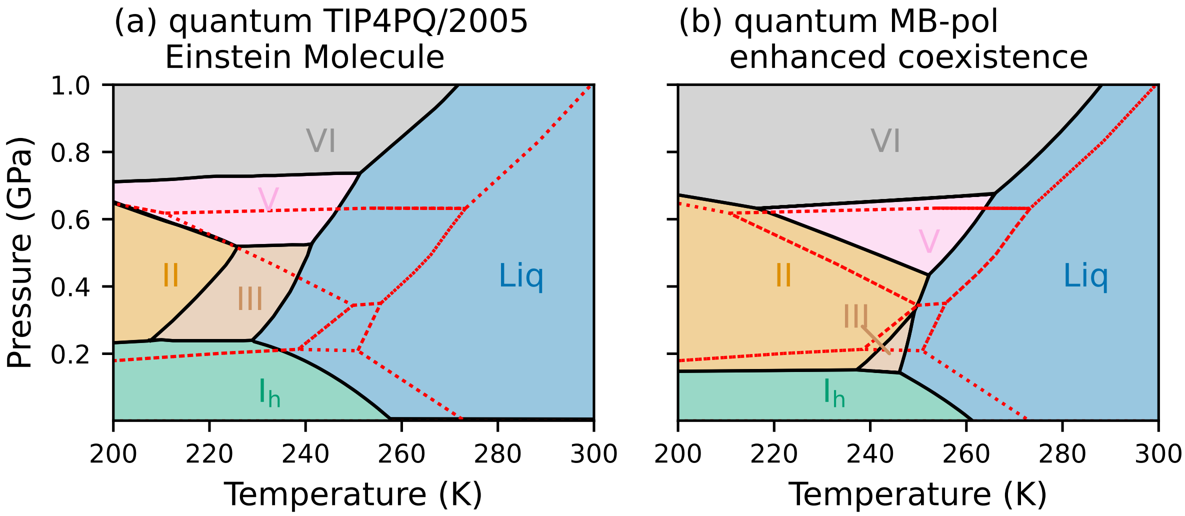

Supplementary Figure 30: **TIP4PQ/2005 vs. MB-pol.** (a) TIP4PQ/2005 phase diagram originally calculated at the quantum level in Ref. 51 using the Einstein Molecule method. As discussed in the text, the actual phase diagram of TIP4PQ/2005 is expected to display a significantly larger area of stability for ice III than that predicted by the Einstein Molecule method. (b) MB-pol phase diagram calculated at the quantum level in this work using enhanced-coexistence simulations.

Supplementary References

1. Babin, V., Leforestier, C. & Paesani, F. Development of a "first principles" water potential with flexible monomers: Dimer potential energy surface, VRT spectrum, and second virial coefficient. *J. Chem. Theory Comput.* **9**, 5395–5403 (2013).

2. Babin, V., Medders, G. R. & Paesani, F. Development of a "first principles" water potential with flexible monomers. II: Trimer potential energy surface, third virial coefficient, and small clusters. *J. Chem. Phys.* **10**, 1599–1607 (2014).

3. Medders, G. R., Babin, V. & Paesani, F. Development of a "first-principles" water potential with flexible monomers. III. Liquid phase properties. *J. Chem. Theory Comput.* **10**, 2906–2910 (2014).

4. Partridge, H. & Schwenke, D. W. The determination of an accurate isotope dependent potential energy surface for water from extensive ab initio calculations and experimental data. *J. Chem. Phys.* **106**, 4618–4639 (1997).

5. Braams, B. J. & Bowman, J. M. Permutationally invariant potential energy surfaces in high dimensionality. *Int. Rev. Phys. Chem.* **28**, 577–606 (2009).

6. Paesani, F. Getting the right answers for the right reasons: Toward predictive molecular simulations of water with many-body potential energy functions. *Acc. Chem. Res.* **49**, 1844–1851 (2016).

7. Reddy, S. K. *et al.* On the accuracy of the MB-pol many-body potential for water: Interaction energies, vibrational frequencies, and classical thermodynamic and dynamical properties from clusters to liquid water and ice. *J. Chem. Phys.* **145**, 194504 (2016).

8. Richardson, J. O. *et al.* Concerted hydrogen-bond breaking by quantum tunneling in the water hexamer prism. *Science* **351**, 1310–1313 (2016).

9. Cole, W. T., Farrell, J. D., Wales, D. J. & Saykally, R. J. Structure and torsional dynamics of the water octamer from THz laser spectroscopy near 215 μm. *Science* **352**, 1194–1197 (2016).

10. Brown, S. E. *et al.* Monitoring water clusters "melt" through vibrational spectroscopy. *J. Am. Chem. Soc.* **139**, 7082–7088 (2017).

11. Medders, G. R. & Paesani, F. Infrared and Raman spectroscopy of liquid water through "first-principles" many-body molecular dynamics. *J. Chem. Theory Comput.* **11**, 1145–1154 (2015).

12. Medders, G. R. & Paesani, F. Dissecting the molecular structure of the air/water interface from quantum simulations of the sum-frequency generation spectrum. *J. Am. Chem. Soc.* **138**, 3912–3919 (2016).

13. Reddy, S. K., Moberg, D. R., Straight, S. C. & Paesani, F. Temperature-dependent vibrational spectra and structure of liquid water from classical and quantum simulations with the mb-pol potential energy function. *J. Chem. Phys.* **147**, 244504 (2017).

14. Moberg, D. R., Straight, S. C. & Paesani, F. Temperature dependence of the air/water interface revealed by polarization sensitive sum-frequency generation spectroscopy. *J. Phys. Chem. B* **122**, 4356–4365 (2018).

15. Muniz, M. C. *et al.* Vapor–liquid equilibrium of water with the MB-pol many-body potential. *J. Chem. Phys.* **154**, 211103 (2021).

16. Gartner III, T. E. *et al.* Anomalies and local structure of liquid water from boiling to the supercooled regime as predicted by the many-body MB-pol model. *J. Phys. Chem* **13**, 3652–3658 (2022).

17. Pham, C. H., Reddy, S. K., Chen, K., Knight, C. & Paesani, F. Many-body interactions in ice. *J. Chem. Theory Comput.* **13**, 1778–1784 (2017).

18. Moberg, D. R., Straight, S. C., Knight, C. & Paesani, F. Molecular origin of the vibrational structure of ice I_h. *J. Phys. Chem. Lett.* **8**, 2579–2583 (2017).

19. Moberg, D. R., Sharp, P. J. & Paesani, F. Molecular-level interpretation of vibrational spectra of ordered ice phases. *J. Phys. Chem. B* **122**, 10572–10581 (2018).

20. Moberg, D. R. *et al.* The End of Ice I. *Proc. Natl. Acad. Sci. U.S.A.* **116**, 24413–24419 (2019).

21. Zhang, L. *et al.* End-to-end symmetry preserving inter-atomic potential energy model for finite and extended systems. In Bengio, S. *et al.* (eds.) *Advances in Neural Information Processing Systems*, vol. 31 (Curran Associates, Inc., 2018).

22. Wang, H., Zhang, L., Han, J. & Weinan, E. DeePMD-kit: A deep learning package for many-body potential energy representation and molecular dynamics. *Comput. Phys. Commun.* **228**, 178–184 (2018).

23. Paesani group (UC San Diego). MBX: An energy and force calculator for data-driven many-body potential energy functions. https://github.com/paesanilab/MBX (2021).

24. Zhai, Y., Caruso, A., Bore, S. L., Luo, Z. & Paesani, F. A "short blanket" dilemma for a state-of-the-art neural network potential for water: Reproducing experimental properties or the physics of the underlying many-body interactions? *J. Chem. Phys.* **158**, 084111 (2023).

25. Pinheiro, M., Ge, F., Ferré, N., Dral, P. O. & Barbatti, M. Choosing the right molecular machine learning potential. *Chem. Sci.* **12**, 14396–14413 (2021).

26. Della Pia, F., Zen, A., Alfè, D. & Michaelides, A. DMC-ICE13: Ambient and high pressure polymorphs of ice from diffusion Monte Carlo and density functional theory. *J. Chem. Phys.* **157**, 134701 (2022).

27. Thompson, A. P. *et al.* LAMMPS – A flexible simulation tool for particle-based materials modeling at the atomic, meso, and continuum scales. *Comput. Phys. Commun.* **271**, 108171 (2022).

28. Tribello, G. A., Bonomi, M., Branduardi, D., Camilloni, C. & Bussi, G. PLUMED 2: New feathers for an old bird. *Comput. Phys. Commun.* **185**, 604–613 (2014).

29. Han, J., Zhang, L., Car, R. *et al.* Deep Potential: A General Representation of a Many-Body Potential Energy Surface. *Commun. Comput. Phys.* **23**, 629–639 (2018).

30. Lu, D. *et al.* DP compress: A model compression scheme for generating efficient deep potential models. *J. Chem. Theory Comput.* **18**, 5559–5567 (2022).

31. Bussi, G., Donadio, D. & Parrinello, M. Canonical sampling through velocity rescaling. *J. Chem. Phys.* **126**, 014101 (2007).

32. Parrinello, M. & Rahman, A. Polymorphic transitions in single crystals: A new molecular dynamics method. *J. Chem. Phys.* **52**, 7182–7190 (1981).

33. Petrenko, V. F. & Whitworth, R. W. *Physics of Ice* (OUP Oxford, 1999).

34. Holten, V., Sengers, J. V. & Anisimov, M. A. Equation of State for Supercooled Water at Pressures up to 400 MPa. *J. Phys. Chem. Ref. Data* **43**, 043101 (2014).

35. Wagner, W. & Pruß, A. The IAPWS Formulation 1995 for the Thermodynamic Properties of Ordinary Water Substance for General and Scientific Use. *J. Phys. Chem. Ref. Data* **31**, 387–535 (2002).

36. Kell, G. S. Isothermal Compressibility of Liquid Water at 1 atm. *J. Chem. Eng. Data* **15**, 119–122 (1970).

37. Speedy, R. & Angell, C. Isothermal compressibility of supercooled water and evidence for a thermodynamic singularity at -45 ^{o}C. *J. Chem. Phys.* **65**, 851–858 (1976).

38. Kim, K. H. *et al.* Maxima in the thermodynamic response and correlation functions of deeply supercooled water. *Science* **358**, 1589–1593 (2017).

39. Bore, S. L., Piaggi, P. M., Car, R. & Paesani, F. Phase diagram of the TIP4P/Ice water model by enhanced sampling simulations. *J. Chem. Phys.* **157**, 054504 (2022).

40. Pedersen, U. R., Hummel, F., Kresse, G., Kahl, G. & Dellago, C. Computing Gibbs free energy differences by interface pinning. *Phys. Rev. B* **88**, 094101 (2013).

41. Piaggi, P. M. Environment finder: A tool for finding and analyzing atomic environments in crystal structures v1.0.1. Zenodo (2021).

42. Piaggi, P. M., Panagiotopoulos, A. Z., Debenedetti, P. G. & Car, R. Phase equilibrium of water with hexagonal and cubic ice using the scan functional. *J. Chem. Theory Comput.* **17**, 3065–3077 (2021).

43. Piaggi, P. M. & Parrinello, M. Calculation of phase diagrams in the multithermal-multibaric ensemble. *J. Chem. Phys.* **150**, 244119 (2019).

44. Bartók, A. P., Kondor, R. & Csányi, G. On representing chemical environments. *Phys. Rev. B* **87**, 184115 (2013).

45. Invernizzi, M. & Parrinello, M. Rethinking metadynamics: from bias potentials to probability distributions. *J. Phys. Chem. Lett.* **11**, 2731–2736 (2020).

46. Invernizzi, M., Piaggi, P. M. & Parrinello, M. Unified approach to enhanced sampling. *Phys. Rev. X* **10**, 041034 (2020).

47. Laio, A. & Parrinello, M. Escaping free-energy minima. *Proc. Natl. Acad. Sci.* **99**, 12562–12566 (2002).

48. Reinhardt, A. & Cheng, B. Quantum-mechanical exploration of the phase diagram of water. *Nat. Commun.* **12**, 588 (2021).

49. Paesani, F. & Voth, G. A. The properties of water: Insights from quantum simulations. *J. Phys. Chem. B* **113**, 5702–5719 (2009).

50. Ceriotti, M. *et al.* Nuclear quantum effects in water and aqueous systems: Experiment, theory, and current challenges. *Chem. Rev.* **116**, 7529–7550 (2016).

51. McBride, C., Noya, E. G., Aragones, J. L., Conde, M. M. & Vega, C. The phase diagram of water from quantum simulations. *Phys. Chem. Chem. Phys.* **14**, 10140–10146 (2012).

52. Cheng, B., Engel, E. A., Behler, J., Dellago, C. & Ceriotti, M. Ab initio thermodynamics of liquid and solid water. *Proc. Natl. Acad. Sci. U.S.A* **116**, 1110–1115 (2019).

53. Ceriotti, M. & Markland, T. E. Efficient methods and practical guidelines for simulating isotope effects. *J. Chem. Phys.* **138**, 014112 (2013).

54. Chandler, D. & Wolynes, P. G. Exploiting the isomorphism between quantum theory and classical statistical mechanics of polyatomic fluids. *J. Chem. Phys.* **74**, 4078–4095 (1981).

55. Berne, B. J. & Thirumalai, D. On the simulation of quantum systems: Path integral methods. *Annu. Rev. Phys. Chem.* **37**, 401–424 (1986).

56. Kofke, D. A. Direct evaluation of phase coexistence by molecular simulation via integration along the saturation line. *J. Chem. Phys.* **98**, 4149–4162 (1993).

57. Dormand, J. R. & Prince, P. J. A family of embedded Runge-Kutta formulae. *J. Comput. Appl. Math.* **6**, 19–26 (1980).

58. Matsumoto, M., Yagasaki, T. & Tanaka, H. GenIce: Hydrogen-Disordered Ice Generator. *J. Comput. Chem.* **39**, 61–64 (2017).

59. Matsumoto, M., Yagasaki, T. & Tanaka, H. Novel Algorithm to Generate Hydrogen-Disordered Ice Structures. *J. Chem. Inf. Model.* **61**, 2542–2546 (2021).

60. Bernal, J. D. & Fowler, R. H. A theory of water and ionic solution, with particular reference to hydrogen and hydroxyl ions. *J. Chem. Phys.* **1**, 515–548 (1933).

61. Frenkel, D. Simulations: The dark side. *Eur. Phys. J. Plus* **128**, 1–21 (2013).

62. Bonomi, M. Promoting transparency and reproducibility in enhanced molecular simulations. *Nat. Methods* **16**, 670–673 (2019).

63. Cheng, B., Behler, J. & Ceriotti, M. Nuclear quantum effects in water at the triple point: Using theory as a link between experiments. *J. Phys. Chem. Lett.* **7**, 2210–2215 (2016).

64. Reinhardt, A. *et al.* Thermodynamics of high-pressure ice phases explored with atomistic simulations. *Nat. Commun.* **13**, 1–10 (2022).

65. Ceriotti, M., Bussi, G. & Parrinello, M. Colored-noise thermostats à la carte. *J. Chem. Theory Comput.* **6**, 1170–1180 (2010).

66. Ceriotti, M., More, J. & Manolopoulos, D. E. i-PI: A Python interface for ab initio path integral molecular dynamics simulations. *Comput. Phys. Commun* **185**, 1019–1026 (2014).

67. Kapil, V. *et al.* i-PI 2.0: A universal force engine for advanced molecular simulations. *Comput. Phys. Commun.* **236**, 214–223 (2019).

68. García Fernández, R., Abascal, J. L. & Vega, C. The melting point of ice I h for common water models calculated from direct coexistence of the solid-liquid interface. *J. Chem. Phys.* **124**, 144506 (2006).

69. Conde, M., Rovere, M. & Gallo, P. High precision determination of the melting points of water TIP4P/2005 and water TIP4P/Ice models by the direct coexistence technique. *J. Chem. Phys.* **147**, 244506 (2017).

70. Rozmanov, D. & Kusalik, P. G. Anisotropy in the crystal growth of hexagonal ice, I_h. *J. Chem. Phys.* **137**, 094702 (2012).

71. Hudait, A., Qiu, S., Lupi, L. & Molinero, V. Free energy contributions and structural characterization of stacking disordered ices. *Phys. Chem. Chem. Phys.* **18**, 9544–9553 (2016).

72. Haji-Akbari, A. & Debenedetti, P. G. Direct calculation of ice homogeneous nucleation rate for a molecular model of water. *Proc. Natl. Acad. Sci. U.S.A.* **112**, 10582–10588 (2015).

73. Conde, M. M., Gonzalez, M., Abascal, J. & Vega, C. Determining the phase diagram of water from direct coexistence simulations: The phase diagram of the TIP4P/2005 model revisited. *J. Chem. Phys.* **139**, 154505 (2013).

74. Abascal, J. L. F. & Vega, C. A general purpose model for the condensed phases of water: TIP4P/2005. *J. Chem. Phys.* **123**, 234505 (2005).

75. Abascal, J. L. F., Sanz, E., García Fernández, R. & Vega, C. A potential model for the study of ices and amorphous water: TIP4P/Ice. *J. Chem. Phys.* **122**, 234511 (2005).
